# Graded exercise therapy compared to activity management for paediatric chronic fatigue syndrome/myalgic encephalomyelitis: pragmatic randomized controlled trial

**DOI:** 10.1007/s00431-024-05458-x

**Published:** 2024-03-02

**Authors:** Daisy M. Gaunt, Amberly Brigden, Shaun R. S. Harris, William Hollingworth, Russell Jago, Emma Solomon-Moore, Lucy Beasant, Nicola Mills, Parisa Sinai, Esther Crawley, Chris Metcalfe

**Affiliations:** 1 Bristol Medical School, Centre for Academic Child Health, University of Bristol, Canynge Hall, 39 Whatley Road, Bristol, BS8 2PS UK; 2 Bristol Medical School, University of Bristol, Canynge Hall, 39 Whatley Road, Bristol, BS8 2PS UK; 3 Bristol Medical School, Bristol Trials Centre, University of Bristol, 1-5 Whiteladies Road, Bristol, BS8 1NU UK; 4 Swansea Centre for Health Economics, Swansea University, Singleton Park, Swansea, SA2 8PP UK; 5 Digital Health, School of Computer Science, Electrical and Electronic Engineering, University of Bristol, Bristol, BS1 5DD UK; 6 Department for Health, University of Bath, Claverton Down, Bath, BA2 7AY UK

**Keywords:** Graded exercise therapy, Activity management, Chronic fatigue syndrome/myalgic encephalomyelitis

## Abstract

The MAGENTA pragmatic parallel groups randomized controlled trial compared graded exercise therapy (GET) with activity management (AM) in treating paediatric myalgic encephalomyelitis or chronic fatigue syndrome (ME/CFS). Children aged 8-17 years with mild/moderate ME/CFS and presenting to NHS specialist paediatric services were allocated at random to either individualised flexible treatment focussing on physical activity (GET, 123 participants) or on managing cognitive, school and social activity (AM, 118 participants) delivered by NHS therapists. The primary outcome was the self-reported short-form 36 physical function subscale (SF-36-PFS) after 6 months, with higher scores indicating better functioning. After 6 months, data were available for 201 (83%) participants who received a mean of 3.9 (GET) or 4.6 (AM) treatment sessions. Comparing participants with measured outcomes in their allocated groups, the mean SF-36-PFS score changed from 54.8 (standard deviation 23.7) to 55.7 (23.3) for GET and from 55.5 (23.1) to 57.7 (26.0) for AM giving an adjusted difference in means of −2.02 (95% confidence interval −7.75, 2.70). One hundred thirty-five participants completed the mean SF-36-PFS at 12 months, and whilst further improvement was observed, the difference between the study groups remained consistent with chance. The two study groups showed similar changes on most of the secondary outcome measures: Chalder Fatigue, Hospital Anxiety and Depression Scale: Depression, proportion of full-time school attended, a visual analogue pain scale, participant-rated change and accelerometer measured physical activity, whether at the 6-month or 12-month assessment. There was an isolated finding of some evidence of an improvement in anxiety in those allocated to GET, as measured by the Hospital Anxiety and Depression Scale at 6 months, with the 12-month assessment, and the Spence Children’s Anxiety scale being aligned with that finding. There was weak evidence of a greater risk of deterioration with GET (27%) than with AM (17%; *p* = 0.069). At conventional UK cost per QALY thresholds, the probability that GET is more cost-effective than AM ranged from 18 to 21%. Whilst completion of the SF-36-PFS, Chalder Fatigue Scale and EQ-5D-Y was good at the 6-month assessment point, it was less satisfactory for other measures, and for all measures at the 12-month assessment.

*Conclusion*: There was no evidence that GET was more effective or cost-effective than AM in this setting, with very limited improvement in either study group evident by the 6-month or 12-month assessment points.

*Trial registration*: The study protocol was registered at www.isrctn.com (3rd September 2015; ISRCTN 23962803) before the start of enrolment to the initial feasibility phase.

**Supplementary Information:**

The online version contains supplementary material available at 10.1007/s00431-024-05458-x.

**Table:** 

**What is Known:** *• Paediatric ME/CFS is disabling with negative effects on physical and cognitive function, mood and quality of life. Although CBT improves fatigue, disability and school attendance, at least 37% of children and young people with ME/CFS have not recovered 6 months after beginning treatment.* *• Graded exercise therapy (delivered flexibly) and activity management are acceptable to children and adolescents with ME/CFS; however, there is no evidence on effectiveness or cost-effectiveness.*	
**What is New:** *• Graded exercise therapy delivered in an out-patient setting is neither more effective nor more cost-effective than activity management for paediatric ME/CFS.* *•Physical function did not improve greatly in either group over the 12 months in this pragmatic study conducted in the UK NHS*	

## Introduction

Paediatric myalgic encephalomyelitis or chronic fatigue syndrome (ME/CFS) has disabling effects on physical function [1], mood [2], quality of life [3–5] and attendance and performance at school [6]. Cognitive behavioural therapy (CBT) improves fatigue, disability and school attendance compared to waiting list or usual medical care [7–11]. However, at least 37% of children have not recovered at 6 months [9]. Alternative approaches, but with limited evidence of effectiveness in the paediatric setting, include graded exercise therapy (GET) and activity management (AM) (also called energy management [12] or pacing).

The MAGENTA study addressed this evidence gap, aiming to test the effectiveness, cost-effectiveness and safety of GET compared to AM for children with mild to moderate ME/CFS.

## Methods

### Study design and participants

MAGENTA was a pragmatic parallel group randomized controlled trial (RCT) recruiting between 10 September 2015 and 23 March 2018, with 12-month follow-up. Recruitment started during an initial feasibility phase [13, 14], which successfully progressed to a full trial (Appendix 1).

Eligible children met the 2007 NICE criteria for a diagnosis of ME/CFS [15], were aged 8 to 17 years, spoke English, were not severely affected [15] and did not require CBT for anxiety or depression at their first clinical assessment. Exclusion criteria included other disorders associated with fatigue [15].

### Randomization and masking

Participants were allocated to GET or AM in a 1:1 ratio using a web-based system (Bristol Trials Centre, UK), accessed by the recruiting nurse. There is minimisation by age (8 to 12 and 13 to 17 years) and sex, weighted towards the allocation minimising the imbalance in trial groups with probability 0.8. This study was necessarily unmasked, with participants, parents and clinicians, aware of allocation.

### Interventions

AM and GET were delivered in 1:1 sessions, face-to-face or via video call. Treatment fidelity was encouraged by training for therapists in each intervention and recording the inclusion of mandatory, prohibited and flexible components of the interventions for each session (Appendix 2).

GET was delivered by specialist therapists as a personalised approach, initially establishing a baseline level of physical activity (walking, sport, etc.) estimated as the median amount of daily physical activity over a week. Children were asked to avoid peaks in exercise, to be able to do the same every day. Participants were offered a detailed assessment of their physical activity at baseline. Once that baseline was established, participants were asked to slowly increase (by 10–20% a week) their physical activity when they felt able to. Participants were asked to monitor exercise and were taught how to stay within 50–70% of their maximum heart rate [13, 14]. If symptoms increased, participants were advised to stabilise or reduce their physical activity.

Activity management (AM) was provided as a personalised approach that established a baseline (similar every day) level of cognitive (e.g. schoolwork, social activities) and physical activity (walking, any exercise) using diaries. This usually required a reduction in activity on some days. Both physical activity and cognitive activity were then gradually increased as participants were able. If participants’ symptoms increased, they were advised to keep activity constant or reduce activity (cognitive and physical). Sessions were delivered by health professionals including specialist doctors, psychologists, physiotherapists, occupational therapists and nurses.

Follow-up sessions were offered to participants in both groups [16]. In this pragmatic trial, the total number and frequency of sessions were agreed between participants and clinicians. From previous paediatric RCTs, we expected between eight and 12 sessions [7, 13, 17]. Participants who developed anxiety or depression after randomization were offered additional CBT.

### Outcomes

Participants completed outcomes measures at baseline, 6 and 12 months. The primary outcome was the SF-36 physical function subscale (SF-36-PFS, scored 0–100 with 100 being the best physical function [18]) collected at 6 months post-randomization. Secondary outcomes were the SF-36-PFS at 12 months, self-reported school attendance (days per week), the Chalder Fatigue scale [19] (11-item version), pain (visual analogue scale with anchors “NO PAIN” and “PAIN AS BAD AS POSSIBLE”), the Hospital Anxiety and Depression Scale (HADS) in those aged 12 and over [20], Spence Children’s Anxiety Scale (SCAS) [21], The Clinical Global Impression Scale [22] and quality of life (EQ-5D-Y [23]). At 6 and 12 months, parents completed an adapted four-item Work Productivity and Activity Impairment: General Health V2.0 (WPAI:GH) questionnaire [24] and a resource use questionnaire. If completed questionnaires had not been received after two reminders, participants were invited to provide just the primary outcome over the phone.

Further secondary outcome measures were derived from data collected by waist-worn accelerometers (Actigraph GT3X+, Actigraph LLC Florida) after randomization, and at 3 and 6 months (Appendix 3) [25]. Minutes of sedentary, light, moderate to vigorous and vigorous intensity activity were derived using accepted cut-offs [26, 27]. Total physical activity volume was derived from accelerometer counts per minute.

Safety outcomes included adverse events, serious adverse events, deterioration in physical function and withdrawal from treatment.

### Sample size

To detect a minimal clinically important between-group difference (MCID) of 10 points (standard deviation 25) on the SF-36-PFS [28], at 6 months, 100 participants in each group were required to provide 80% power at 5% significance. A target of 230 participants allowed for missing data.

### Statistical analysis

The statistical and health economic analysis plan was published online in November 2019 (Appendix 4), prior to the senior authors having sight of the data. The primary analysis compared mean SF-36-PFS score, analysed as a continuous variable, at 6 months according to random allocation (intention-to-treat) among participants with measured outcomes, using multivariable linear regression adjusting for baseline values of the outcome, age and gender. Similar analyses were conducted for secondary outcomes.

A pre-specified subgroup analysis compared GET and AM in males and females separately. Sensitivity analyses for the primary outcome were additional adjustment by the number of days between random allocation and primary outcome measure completion and the proportion of school attended at baseline, and multiple imputation of missing outcome data under the missing at random assumption. A pre-specified analysis examined the relationship between the treatment effect and the number of sessions attended.

### Health economic analyses

A cost-utility analysis compared GET versus AM from the health service and public sector perspective. We estimated the incremental net monetary benefit (iNMB) of GET, at a threshold willingness-to-pay of £20,000 (~ US$30,000) per QALY [29].

Training and supervision costs for healthcare professionals were assumed to be equal for GET and AM. GET and AM outpatient sessions and additional appointments for CBT were extracted from hospital records. Other healthcare use was based on parent-report. We assumed that, on average, sessions associated with either GET or AM lasted for 60 min. Resource use was valued using 2019 unit costs [30, 31].

Quality-of-life preference scores derived from the EQ-5D-Y visual analogue scale (VAS) were converted to a 0–1 scale and QALYs calculated using linear interpolation to estimate the area under the curve. We used the adult value set for the EQ-5D-Y [32] to estimate preference scores and QALYs in a sensitivity analysis. Multiple imputation (*n* = 50 imputations) was used for missing EQ-5D-Y and total cost data (Appendix 3). Incremental costs, QALYs and net benefits were estimated using seemingly unrelated regression adjusting for age, sex and baseline values of the dependent variable. Non-parametric bootstrap 95% confidence intervals were employed for the iNMB. The probability that GET was more cost-effective than AM across a range of cost per QALY thresholds was investigated using the cost-effectiveness acceptability curve method [33].

All analyses used Stata version 15.1 (StataCorp, College Station, Texas).

## Results

**Fig. 1:**
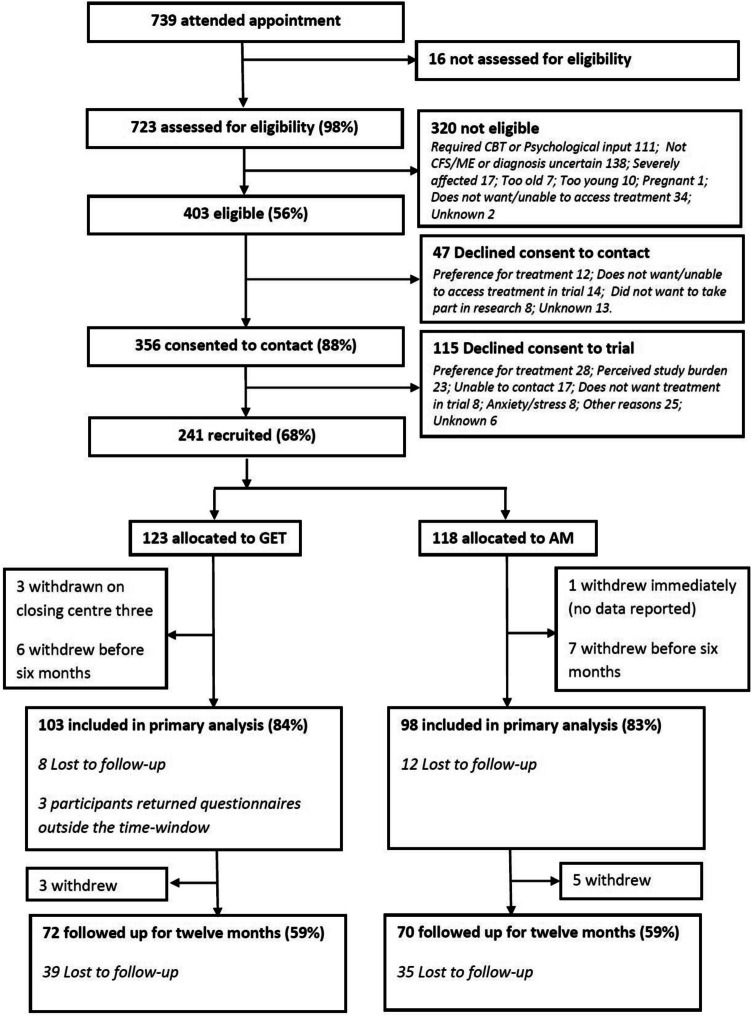
CONSORT recruitment and retention flow chart

MAGENTA closed as planned on achieving its recruitment target, with 241 children (60% of eligible patients) randomly allocated to GET or AM (Fig. 1). Participants’ characteristics at baseline were balanced between treatment groups (Table 1). Participants were disabled by their fatigue with only 28 (12%) attending full-time school, although some children reported having high physical function at baseline (Supplementary Fig. 1).

**Table 1:** Baseline characteristics of participants allocated to graded exercise therapy (GET) or activity management (AM)

	**GET (*n* = 123)**		**AM (*n* = 117**^**a**^**)**	
		*n*		*n*
Mean age (SD)	14.3 (2.2)	123	14.2 (2.3)	117
Number female (%)	89 (72%)	123	87 (74%)	117
Median months since illness onset (25th, 75th percentiles)	14 (10, 24)	120	15 (10, 24)	117
Mean SF-36 Physical Function (SD)	54.4 (24.2)	119	54.8 (23.7)	115
Mean Chalder Fatigue score (SD)	24.5 (5.0)	119	24.0 (4.9)	115
Mean pain VAS (SD)	45.3 (26.7)	110	44.6 (27.6)	103
Mean SCAS (SD)	35.1 (18.1)	118	32.3 (19.4)	116
Mean HADS anxiety score (SD)	9.5 (4.4)	103	8.2 (4.4)	102
Mean HADS depression score (SD)	7.9 (3.9)	103	7.0 (3.6)	102
*School attendance in previous week:*				
None (%)	14 (13%)	111	16 (14%)	115
About 10%/half day (%)	6 (5%)		13 (11%)	
About 20%/1 day (%)	7 (6%)		7 (6%)	
About 40%/2 days (%)	19 (17%)		15 (13%)	
About 60%/3 days (%)	25 (23%)		18 (16%)	
About 80%/4 days (%)	25 (23%)		33 (29%)	
Full time/about 5 days (%)	15 (14%)		13 (11%)	
*Accelerometer data:*				
Counts per minute (SD)	280.5 (165.5)	84	309.9 (266.3)	82
*Mean daily minutes in the following physical activity states:*				
Sedentary (SD)	578.3 (145.2)	84	593.9 (172.2)	82
Light intensity (SD)	134.4 (53.5)	84	136.8 (52.2)	82
Moderate-to-vigorous intensity (SD)	28.7 (21.1)	84	28.7 (23.9)	82
Vigorous intensity (SD)	10.3 (12.7)	84	9.1 (11.4)	82

a. One participant dropped out immediately after random allocation and provided no data

Adherence was high, with 114 (96%) and 109 (94%) starting their allocated GET and AM intervention respectively (Table 2; Supplementary Fig. 2). Thirteen participants started non-allocated treatment before the primary outcome was measured at 6 months: five in AM and five in GET switched to the other study treatment; two in GET started CBT only and one in AM added GET to their AM treatment. Fidelity in allocated treatment sessions was high with three GET and nine AM participants receiving a prohibited element or not receiving a compulsory element during their allocated treatment. CBT for participants who developed depression or anxiety was received by 32 (27%) participants in the GET group and 27 (23%) participants in the AM group, with a mean of 5.5 and 5.4 sessions over 12 months respectively.

**Table 2:** Treatment fidelity and adherence for participants allocate to graded exercise therapy (GET) or activity management (AM)

	**GET (*n* = 119)**	**AM (*n* = 116)**
Number of participants not attending any sessions of treatment (%)	5 (4%)	7 (6%)
Number starting non-allocated treatment within six months of randomization (%)	7 (6%)	6 (5%)
Number starting non-allocated treatment between six and twelve months of randomization (%)	7 (6%)	7 (6%)
Number of participants with one or more sessions recorded as not including a compulsory element or including a prohibited element (%)	3 (3%)	9 (8%)
Number completing treatment (≥ 8 sessions) within six months of randomization (%)	4 (3%)	11 (9%)
**Of those completing at least one session of allocated treatment:**		
Mean (SD) sessions of treatment within six months of randomization	3.9 (1.9)	4.6 (2.1)
Mean (SD) sessions of treatment over 12 months of follow-up	6.2 (3.2)	6.9 (3.5)

Data available for the 235 participants from Centre One

The mean total number of allocated treatment sessions was 3.9 in the GET group and 4.6 in the AM group by 6 months (Table 2; Supplementary Fig. 2). The mean (SD) time between baseline clinical assessment and primary outcome collection at the 6-month assessment point was 7.4 (1.4) and 7.3 months (1.5) in GET and AM respectively, with data available for 201 (83%) participants. Compared to baseline measures, only small improvements in the mean SF-36-PFS scores were observed at the 6-month assessment (the primary outcome) in both study groups, with no evidence of a difference between the groups (adjusted difference in means −2.02, 95% confidence interval −7.75 to 3.70, *p* = 0.49; Table 3). Sensitivity analyses supported the same conclusions (Supplementary Table 1). There was no evidence that those participants attending more than two sessions saw their physical function improve by the 6-month assessment point (Supplementary Table 1). These results are lower than the current estimated MCID of 10 points on the SF-36-PFS used in the estimation of the sample size for this trial [28].

**Table 3:** Summary statistics and treatment effect estimates for the SF-36 physical function subscale (SF-36-PFS) at 6 months (primary outcome measure) and 12 months, comparing participants as allocated to graded exercise therapy (GET) and activity management (AM)

	**GET**	**AM**	**Adjusted difference in means (95% CI)**^a^	***p* value**
	**Mean (SD), *N***	**Mean (SD), *N***		
**SF-36-PF at 6 months (primary outcome)**				
6-month scores	55.7 (23.3), 103	57.7 (26.0), 98	−2.02 (−7.75, 3.70)	0.49
Baseline scores^b^	54.8 (23.7), 101	55.5 (23.1), 96		
**Pre-specified subgroup analysis, SF-36-PF at 6 months**				
Females	55.5 (21.4), 75	53.3 (25.6), 73	1.06 (−5.46, 7.58)	Interaction p-value
Males	57.7 (27.1), 31	70.4 (23.4), 25	−11.17 (−23.35, 1.01)	0.71
**SF-36-PF at 12 months (secondary outcome)**				
12-month scores	60.9 (23.5), 68	59.2 (29.8), 67	3.15 (−4.20, 10.51)	0.40
Baseline scores^c^	52.9 (24.4), 66	55.1 (23.9), 65		

Higher score = fewer symptoms, better function

^a^Multivariable linear regression adjusting for baseline values of the outcome, baseline age and sex and an indicator variable denoting whether the baseline assessment of the outcome measure was observed

^b^For those also completing the 6-month assessment

^c^For those also completing the 12-month assessment

There was no evidence (interaction *p*-value 0.71) of a difference in the relative effects of GET and AM between males and females (Table 3). Modest increases in mean scores were observed in those participants completing the SF-36-PFS at the 12-month assessment, with no evidence of a difference between the two allocated groups (Table 3).

There was little evidence of differences between groups in the secondary outcome measures (Supplementary Table 2). The allocated groups saw similar improvements over time in mean Chalder Fatigue scores. Fewer participants responded to the other secondary measures, with an overall picture of similarly modest improvements over 12 months in the two allocated groups. The mean number of allocated treatment sessions attended by the 12-month assessment was 6.2 for participants allocated to GET and 6.9 for those allocated to AM (Table 2). There was some evidence from the HADS completed at 6 months of a greater improvement in anxiety for participants allocated to GET compared to AM. School attendance was unchanged at 6- and 12 months in both treatment groups. Overall, 30% (26% GET, 34% AM) felt they were much better or very much better using the CGI (Supplementary Table 3) at 6 months and 40% at 12 months. For the minority of participants who returned accelerometer data, derived counts per minute were similar between both treatment groups at baseline, 3 and 6 months (Supplementary Table 4).

Five participants in both study groups reported being much worse or very much worse when completing the CGI at the 6-month assessment (Supplementary Table 3) with similar findings at 12 months. Physical function between baseline and 6 or 12 months deteriorated by 20 points in 18 of 97 participants (19%) allocated to GET and 24 of 104 participants (23%) allocated to AM. Four serious adverse events requiring hospital treatment were reported (Supplementary Table 5): one event in each of two participants allocated to AM, two events in one participant allocated to GET. One serious adverse event was possibly related to GET, a psychiatric hospital admission due to suicidal ideation. Five participants withdrew from their allocated therapy due to feeling worse, four of 117 (3%) in the GET group and 1/123 (1%) participants in the AM group. There were no additional clinician reports of serious deteriorations in the study participants. Combining these measures, there was evidence of deterioration (from at least one measure) in 33 out of 123 (27%) of participants in the GET group and 20 out of 117 (17%) participants in the AM group (*p* = 0.069).

Complete healthcare use questionnaires were returned by parents regarding 124 participants (51%) at 6 months and 115 (48%) participants at 12 months. Participants assigned to GET had more therapy delivered by a physiotherapist, whereas participants allocated to AM were more likely to get care delivered by a psychologist or occupational therapist (Supplementary Table 6). Considering all healthcare professionals, participants allocated to AM attended a mean of 7.74 appointments with healthcare professionals over 12 months compared to 6.90 for participants allocated to GET. The average cost per participant of specialist care over 12 months was £46 (95% CI, − £5, £97) more expensive for participants allocated to AM (Supplementary Table 5 with further detail of costs in Supplementary Table 7).

EQ-5D scores demonstrated small improvements in both groups over the 12-month follow up (Supplementary Table 8). There was no evidence of a difference in QALYs (−0.02; 95% CI −0.05 to 0.01; Table 4) between participants randomized to GET and AM. At 12 months, the incremental net monetary benefit of GET compared to AM was − £343 (95% CI, − £1183, £497) at a £20,000 per QALY threshold. At willingness-to-pay thresholds of £20,000 and £30,000 per QALY, the probability that GET is more cost-effective than AM at 12 months was 21% and 18% respectively (Table 4; Supplementary Fig. 3). The sensitivity analysis using adult value sets to estimate QALYs provided similar results (Supplementary Table 9).

**Table 4:** Cost-Effectiveness comparing cost-effectiveness between participants allocated to graded exercise therapy (GET) or activity management (AM)

	**GET (*n* = 123)** Mean (95% CI)	**AM (*n* = 117**^a^**)** Mean (95% CI)	**Adjusted difference (95% CI)**
EQ-5D-Y VAS QALY	0.50 (0.48, 0.53)	0.52 (0.50, 0.54)	−0.02 (−0.05, 0.01)
Cost (£)	1735.08 (1407.22, 2062.95)	1724.63 (1394.38, 2054.88)	10.45 (−465.62, 486.53)
INMB at £20,000 (£)^b^			−342.57 (−1182.60, 497.46)
CE %			21.00%
INMB at £30,000 (£)^2^			−516.80 (−1647.67, 614.06)
CE %			18.31%

^a^One participant dropped out immediately after random allocation and provided no data

^b^The negative INMB implies that GET is unlikely to be cost-effective compared to AM. GET was marginally more expensive and less effective therefore it is not appropriate to report the incremental cost effectiveness ratio

## Discussion

There was little difference in physical function after 6 months between children in the two allocated groups. On average, physical function did not improve to a clinically significant degree (which we have previously determined to be a MCID of 10 points [28]) in either group after 6 or 12 months, consistent with the accelerometer data which suggests the MAGENTA participants had a reduction in moderate-to-vigorous-intensity physical activity at 3 and 6 months. Some outcomes did change: fatigue improved in both groups, sustained to 12 months, which may explain why overall, 40% felt they were much better or very much better after a year. Whilst the observed incremental net monetary benefit at a £20,000 per QALY threshold favoured AM, this estimated difference between the two interventions was imprecise and could have arisen by chance. These results must be interpreted in the light of the pragmatic nature of the study in which, for the majority of participants, sessions took place over 12 months with up to 6-week intervals, reflecting the reality of delivering interventions for paediatric ME/CFS within the NHS. Few adverse events occurred, suggesting that both treatments were safe.

The lack of improvement in self-reported physical function in either group was unexpected, contrasting with the findings of adult treatment trials using the SF-36-PFS measure [34] and our earlier SMILE RCT of interventions for paediatric ME/CFS. The comparison group for the SMILE study was AM with additional GET or CBT when required, which saw a mean improvement between baseline and 6 months of 14 points on the SF-36-PFS [35]. Pragmatic RCTs, like MAGENTA, most commonly aim to establish whether the intervention under evaluation would offer improvements, in terms of increased effectiveness and avoidance of treatment harms, in comparison with the best available alternative used in routine clinical practice, with this approach also avoiding the ethical concerns of asking children to forego a routinely available treatment to join a no-treatment control group. The key strengths of MAGENTA were its randomized design, its acceptability with a majority of eligible patients willing to participate, the pragmatic approach with participants receiving treatments as delivered in the NHS and the high completion of the primary outcome. MAGENTA does have several limitations however. As many participants attended sessions of their allocated intervention over a 12-month period, pre-defining the primary outcome at the 12-month assessment may have better captured the full effects of GET and AM. Only cautious conclusions can be drawn from those secondary outcome measures with poor completion rates. Whilst we registered MAGENTA before starting recruitment during the feasibility phase, we did not confirm the primary outcome measure until the first full trial protocol (Appendices 1 and 5); we recognise this invites the accusation that we used the data collected during the feasibility phase to select the primary outcome. Furthermore, the economic evaluation has been conducted from a NHS perspective only and excludes societal and productivity costs. Finally, in October 2021, NICE revised its ME/CFS diagnostic criteria for technology appraisal [12]. We cannot retrospectively determine if the MAGENTA participants meet these new diagnostic criteria, which have been criticised [36]. We believe, however, that the MAGENTA findings remain relevant evidence to inform the treatment of children presenting with ME/CFS.

## Conclusions

We did not show a difference between GET and AM, or a substantial improvement in physical function with either intervention. This lack of improvement in physical function may be explained by the low intensity of therapy sessions.

### Supplementary Information

Below is the link to the electronic supplementary material.

Supplementary file1 (DOCX 4153 KB)

Supplementary file2 (DOCX 21 KB)

Supplementary file3 (DOCX 27 KB)

Supplementary file4 (DOCX 18 KB)

Supplementary file5 (DOCX 88 KB)

Supplementary file6 (PDF 1770 KB)

## Data Availability

The study participants provided consent to their data being retained and used by the University of Bristol for present and future research and teaching purposes. Individual study data cannot be released to research groups outside of the University of Bristol. Trial registration: MAGENTA is registered at https://www.isrctn.com/ISRCTN23962803, and the pre-specified Statistical Analyses Plan has been publicly available since 14/11/2019: https://research-information.bris.ac.uk/en/publications/magenta-managed-activity-graded-exercise-in-teenagers-and-pre-ado

## Declarations

### Disclaimer

This report is independent research, and the views expressed in this publication of are those of the authors and not necessarily those of the UK National Institute for Health and Care Research, or the UK Department of Health & Social Care. The funder had no role in the collection, analysis, interpretation of data, writing of the report or the decision to submit the article for publication.
