# Supplementary material for: Graded exercise therapy compared to activity management for paediatric chronic fatigue syndrome/myalgic encephalomyelitis: pragmatic randomized controlled trial
Source: Eur J Pediatr. 2024 Mar 2;183(5):2343–51. doi: 10.1007/s00431-024-05458-x (PMC11035451; doi:10.1007/s00431-024-05458-x)
Supplement: Supplementary file 1 — Supplementary file1 (DOCX 4153 KB) [file 431_2024_5458_MOESM1_ESM.docx]

**Graded Exercise Therapy compared to Activity Management for paediatric Chronic Fatigue Syndrome / Myalgic Encephalomyelitis: pragmatic randomized controlled trial**

Daisy M Gaunt*^1 2 3^ (MSc), Amberly Brigden*^5^ (PhD), Shaun R S Harris^4^ (PhD), William Hollingworth^2^ (PhD), Russell Jago^2^ (PhD), Emma Solomon-Moore^6^ (PhD), Lucy Beasant^1^ (PhD), Nicola Mills^2^ (PhD), Parisa Sinai^1^ (PhD), Esther Crawley^1^ (PhD), Chris Metcalfe^2^ ^3^ (PhD)

European Journal of Pediatrics

**Supplementary Tables and Figures**

**Supplementary Table 1.** Sensitivity analysis of SF-36 Physical Function Subscale (SF-36-PFS) at six months in participants as allocated to Graded Exercise Therapy (GET) and Activity Management (AM)

| **SF-36-PFS** | **GET**  Mean (SD), n | **AM**  Mean (SD), n | **Difference in means (95% CI)** | **p-value** |
| --- | --- | --- | --- | --- |
| Estimate from primary analysis (from Table 3) |  |  | -2.02 (-7.75, 3.70) | 0.49 |
| Adjusted by additional covariates^a^ |  |  | -2.03 (-8.03, 3.98) | 0.51 |
| Multiple imputed data  (n=236) |  |  | -2.42 (-8.91,4.06) | 0.46 |
|  | | |  |  |
| Participants attending ≤2 treatment sessions | | |  |  |
| 6 months assessment | 63.6 (22.7), 18 | 76.0 (19.6), 10 | -9.77 (-25.7, 6.2) | 0.22 |
| Baseline assessment | 44.4 (20.0), 17 | 58.3 (23.7), 9 |  |  |
|  | | |  |  |
| Participants attending 3+ treatment sessions | | |  |  |
| 6 months assessment | 54.1 (23.3), 84 | 55.7 (26.0), 87 | -2.67 (-8.68, 3.33) | 0.38 |
| Baseline assessment | 57.1 (24.0), 83 | 55.6 (23.1), 86 |  |  |
|  |  |  |  |  |

a. Adjusted by number of days post-randomization on which primary outcome measure was completed, and proportion of school attendance at baseline (response not applicable treated as missing)

**Supplementary Table 2.** Summary statistics and treatment effect estimates for the secondary outcomes at six and 12 months, for participants as allocated to Graded Exercise Therapy (GET) or Activity Management (AM)

|  | **GET** | **AM** | **Difference in means (95% CI)** | **p-value** |
| --- | --- | --- | --- | --- |
|  | Mean (SD), n | Mean (SD), n |  |  |
| **Chalder Fatigue^a^** | |  |  |  |
| Baseline^b^ | 24.7 (4.8), 101 | 23.9 (4.5), 96 | -- | -- |
| 6 months | 19.9 (7.2), 103 | 19.7 (7.1), 98 | -0.21 (-2.11, 1.69) | 0.83 |
| 12 months | 19.2 (7.5), 69 | 18.5 (7.9), 67 | 0.49 (-2.05, 3.02) | 0.71 |
| **Pain Visual Analogue Scale^a^** | |  |  |  |
| Baseline^b^ | 41.7 (30.1), 42 | 46.2 (27.2), 54 | -- |  |
| 6 months | 38.6 (30.0), 46 | 39.3 (27.4), 60 | 3.83 (-4.76, 12.42) | 0.38 |
| 12 month | 35.5 (27.3), 43 | 40.1 (27.6), 41 | -1.69 (-12.39, 9.01) | 0.75 |
| **Spence Children’s Anxiety Scale^a^** | | |  |  |
| Baseline^b^ | 36.2 (21.0), 45 | 31.3 (19.0), 58 | -- | -- |
| 6 months | 32.4 (18.1), 46 | 34.1 (21.6), 59 | -4.52(-10.33, 1.28) | 0.13 |
| 12 months | 28.3 (20.3), 43 | 32.1 (20.2), 41 | -2.73 (-9.25, 3.78) | 0.41 |
| **Hospital Anxiety & Depression Scale^a^ anxiety** | | |  |  |
| Baseline^b^ | 9.7 (4.6), 40 | 8.2 (4.3), 52 | -- | -- |
| 6 months | 8.1 (4.5), 42 | 9.6 (4.9), 54 | -2.05 (-3.80, -0.30) | 0.02 |
| 12 months | 7.6 (4.8), 40 | 8.0 (4.8), 37 | -0.65 (-2.55,1.25) | 0.50 |
| **Hospital Anxiety & Depression Scale^a^ depression** | | | |  |
| Baseline^b^ | 9.7 (4.6), 40 | 8.2 (4.3), 52 | -- | -- |
| 6 months | 6.3 (3.5), 42 | 7.1 (4.5), 54 | -1.17 (-2.68, 0.34) | 0.13 |
| 12 months | 5.2 (3.5), 39 | 6.0 (4.7), 37 | -0.77 (-2.43, 0.90) | 0.36 |
| **Proportion of full-time school attended^c^** | | |  |  |
| Baseline^b^ | 0.5 (0.3), 56 | 0.5 (0.3), 74 | -- | -- |
| 6 months | 0.5 (0.4), 62 | 0.5 (0.3), 76 | -0.01 (-0.10, 0.08) | 0.78 |
| 12 months | 0.6 (0.4), 47 | 0.5 (0.3), 42 | 0.01(-0.13, 0.15) | 0.91 |

a. Higher score=more symptoms, poorer function

b. Baseline score for those who completed at 6-months

c. Those who responded as not applicable are excluded. Higher score=more school

**Supplementary Table 3.** Participant-rated Clinical Global Impression Scale of change in overall health from baseline, for participants allocated to Graded Exercise Therapy (GET) or Activity Management (AM)

|  | **GET** | **AM** | **Odds ratio**  **(95% CI)^a^** | **p-value** |
| --- | --- | --- | --- | --- |
| **Six months** | **n=85** | **n=86** |  |  |
| Much better or very much better (%) | 22 (26%) | 29 (34%) | -- |  |
| Minimal change (%)^b^ | 58 (68%) | 52 (60%) | -- |  |
| Much worse or very much worse (%) | 5 (6%) | 5 (6%) | 1.33 (0.77, 2.32) | 0.31 |
|  |  |  |  |  |
| **12 months** | **n=59** | **n=53** |  |  |
| Much better or very much better (%) | 22 (37%) | 23 (43%) | -- |  |
| Minimal change (%)^b^ | 34 (58%) | 25 (47%) | -- |  |
| Much worse or very much worse (%) | 3 (5%) | 5 (9%) | 1.37 (0.69, 2.73) | 0.37 |

a. Using ordered logistic regression.

b. Includes the responses “no change”, “a little better”, and “a little worse”.

**Supplementary Table 4.** Summary statistics and treatment effect estimates for the accelerometer measures at 3 months and 6 months for participants as allocated to Graded Exercise Therapy (GET) or Activity Management (AM)

|  | **GET** | **AM** | **Difference in means**  **(95% CI)** | **p-value** |
| --- | --- | --- | --- | --- |
|  | Mean (SD), N | Mean (SD), N |  |  |
| **Counts per minute** | |  |  |  |
| Baseline^a^ | 297.7 (191.1), 27 | 267.8 (148.1), 30 | -- | -- |
| 3 months | 261.7 (103.1), 42 | 273.3 (137.3), 46 | -1.27 (-49.77, 47.23) | 0.96 |
| 6 months | 272.5 (131.2), 31 | 283.9 (151.9), 35 | -26.79 (-88.97, 35.39) | 0.39 |
| **Minutes per day sedentary** | | |  |  |
| Baseline^a^ | 597.7 (167.2), 27 | 622.3 (186.6), 30 | -- | -- |
| 3 months | 567.8 (146.2), 42 | 617.3 (245.9), 46 | -60.52 (-150.27, 29.24) | 0.18 |
| 6 months | 597.1 (227.2), 31 | 596.2 (205.1), 35 | -2.16 (-104.98, 100.66) | 0.97 |
| **Minutes per day light intensity activity** | | |  |  |
| Baseline^a^ | 137.7 (52.2), 27 | 141.0 (51.1), 30 | -- | -- |
| 3 months | 132.3 (46.4), 42 | 137.6 (57.4), 46 | -0.20 (-19.63, 19.23) | 0.98 |
| 6 months | 133.8 (59.4), 31 | 141.3 (62.9), 35 | -11.42 (-38.14, 15.30) | 0.40 |
| **Minutes per day moderate-to-vigorous intensity activity** | | | |  |
| Baseline^a^ | 30.1 (21.3), 27 | 28.6 (21.0), 30 |  |  |
| 3 months | 14.5 (9.2), 42 | 15.3 (11.2), 46 | -1.03 (-5.30, 3.25) | 0.63 |
| 6 months | 17.2 (10.9), 31 | 19.0 (12.4), 35 | -2.27 (-7.64, 3.11) | 0.40 |
| **Minutes per day vigorous intensity activity** | | |  |  |
| Baseline^a^ | 11.7 (15.6), 27 | 6.9 (7.1), 30 |  |  |
| 3 months | 8.2 (7.2), 42 | 8.5 (8.6), 46 | -0.85 (-3.65, 1.94) | 0.55 |
| 6 months | 8.5 (8.0), 31 | 8.9 (8.0), 35 | -1.75 (-5.34, 1.85) | 0.34 |

a. Baseline score for those who also provided data at six months

**Supplementary Table 5.** Brief descriptions of adverse events and serious adverse events.

| Allocation | Participant | AE or SAE | Event description |
| --- | --- | --- | --- |
| GET | A | AE | Worsening of CFS/ME symptoms |
| GET | B | AE | Worsening of CFS/ME symptoms and mood |
| GET | C | AE | Chest pain & difficulty breathing, reassured by clinical staff |
| GET | C | SAE | Hospital admission following self-harm |
| GET | C | SAE | Hospital admission following suicidal ideation |
| AM | D | SAE | Hospital admission following self-harm |
| AM | E | SAE | Cancer diagnosis |

**Supplementary Table 6** Therapy appointments attended (from hospital record review) and associated costs for participants as allocated to Graded Exercise Therapy (GET) or Activity Management (AM)

|  |  | **GET (N=119)** | | | **AM (N=116)** | | |
| --- | --- | --- | --- | --- | --- | --- | --- |
| Clinician type | Period | n^a^ | Total contacts | Mean (95% CI)  per patient | n^a^ | Total contacts | Mean (95% CI) per patient |
| Doctor | 0-6M | 5 | 5 | 0.04 (0.01, 0.08) | 14 | 24 | 0.21 (0.08, 0.34) |
|  | 6-12M | 12 | 13 | 0.11 (0.05, 0.17) | 13 | 13 | 0.11 (0.05, 0.17) |
| Physiotherapist | 0-6M | 94 | 320 | 2.69 (2.34, 3.04) | 36 | 116 | 1.00 (0.67, 1.33) |
|  | 6-12M | 63 | 170 | 1.43 (1.13, 1.73) | 31 | 77 | 0.66 (0.40, 0.93) |
| Psychologist | 0-6M | 18 | 38 | 0.32 (0.15, 0.49) | 43 | 208 | 1.79 (1.28, 2.31) |
|  | 6-12M | 14 | 34 | 0.29 (0.11, 0.47) | 27 | 70 | 0.60 (0.36, 0.84) |
| Occupational Therapist | 0-6M | 17 | 52 | 0.44 (0.23, 0.64) | 37 | 138 | 1.19 (0.83, 1.55) |
|  | 6-12M | 16 | 47 | 0.39(0.20, 0.59) | 30 | 85 | 0.73 (0.48, 0.99) |
| Nurse | 0-6M | 7 | 24 | 0.20 (0.04, 0.36) | 4 | 15 | 0.13 (-0.05, 0.31) |
|  | 6-12M | 4 | 9 | 0.08 (0.00, 0.15) | 1 | 7 | 0.06 (-0.06, 0.18) |
| CBT | 0-6M | 20 | 69 | 0.58 (0.30, 0.86) | 18 | 64 | 0.55 (0.28, 0.82) |
|  | 6-12M | 26 | 108 | 0.91 (0.54, 1.28) | 22 | 81 | 0.70 (0.37, 1.02) |
|  |  |  |  |  |  |  |  |
| Total contacts  (excl. CBT) | 0-6M | 114 |  | 3.85 (3.50, 4.20) | 110 |  | 4.55 (4.14, 4.97) |
|  | 6-12M | 94 |  | 2.90 (2.50, 3.30) | 89 |  | 2.83 (2.44, 3.23) |
|  | 0-12M | 94 |  | 6.90 (6.26, 7.55) | 89 |  | 7.74 (7.06, 8.42) |
|  |  |  |  |  |  |  |  |
| Total contact costs (excl. CBT)^b^, £ | 0-6M | 114 |  | 211.59  (192.35, 230.83) | 110 |  | 250.36  (227.76, 272.96) |
|  | 6-12M | 94 |  | 159.64  (137.67, 181.61) | 89 |  | 155.65  (133.93, 177.38) |
|  | 0-12M | 94 |  | 379.39  (344.04, 414.75) | 89 |  | 425.57  (388.25, 462.90) |

a. The number of participants with 1 or more appointment

b. Total cost of contacts for allocated intervention only, excluding any cognitive behavioural therapy

**Supplementary Table 7: Total unadjusted mean resource use by allocation arm^a^**

| **Resource Use Category: NHS and PSS (unit of measurement)** | **Activity Management** | | **Graded Exercise Therapy** | |
| --- | --- | --- | --- | --- |
|  | **N** | **Mean Resource Use**  **(95% CI)** | **N** | **Mean Resource Use**  **(95% CI)** |
| Inpatient costs (baseline) | 25 | 0.00  (0.00, 0.00) | 22 | 0.00  (0.00, 0.00) |
| Inpatient costs (6-months) | 63 | 42.61  (-17.13, 102.34) | 61 | 22.00  (-22.01, 66.01) |
| Inpatient costs (12-months) | 61 | 44.00  (-17.72, 105.72) | 53 | 0.00  (0.00, 0.00) |
| Outpatient costs (baseline) | 22 | 313.76  (229.09, 398.42) | 25 | 284.47  (210.20, 358.74) |
| Outpatient costs (6-months) | 39 | 750.87  (521.87, 979.87) | 46 | 686.62  (505.80, 867.44) |
| Outpatient costs (12-months) | 31 | 728.72  (432.98, 1024.46) | 34 | 769.01  (446.24, 1091.78) |
| Accident and Emergency costs (baseline) | 25 | 0.00  (0.00, 0.00) | 22 | 11.81  (-5.14, 28.75) |
| Accident and Emergency costs (6-months) | 26 | 19.98  (0.68, 39.28) | 26 | 34.97  (-10.90, 80.84) |
| Accident and Emergency costs (12-months) | 14 | 9.28  (-10.77, 29.32) | 11 | 11.81  (-14.50, 38.12) |
| Primary care costs (baseline) | 25 | 62.65  (13.68, 111.63) | 22 | 54.03  (-43.17, 151.23) |
| Primary care costs (6-months) | 63 | 74.20  (34.16, 114.24) | 61 | 76.99  (12.27, 141.71) |
| Primary care costs (12-months) | 61 | 36.76  (11.64, 61.90) | 54 | 35.83  (-4.87, 76.53) |
| Medication costs (baseline) | 25 | 6.75  (1.69, 11.81) | 22 | 1.93  (0.23, 3.63) |
| Medication costs (6-months) | 63 | 5.87  (0.82, 10.92) | 61 | 3.82  (1.94, 5.69) |
| Medication costs (12-months) | 61 | 7.72  (1.51, 13.93) | 54 | 3.72  (1.85, 5.59) |
| Therapy costs (0 to 6 months) | 116 | 237.41  (213.68, 261.15) | 119 | 202.70  (182.72, 222.68) |
| Therapy costs (6 to 12 months) | 116 | 119.42  (98.86, 139.99) | 119 | 126.10  (105.14, 147.06) |

1. Productivity costs have been excluded from the analysis

**Supplementary Table 8.** EQ-5D scores (available cases) for participants as allocated to Graded Exercise Therapy (GET) or Activity Management (AM)

|  |  | **GET** | |  | **AM** | |
| --- | --- | --- | --- | --- | --- | --- |
|  | Period | N | Mean (CI 95%) |  | N | Mean (CI 95%) |
| EQ-5D-Y VAS^a^ | Baseline | 123 | 0.48 (0.45, 0.51) |  | 117 | 0.49 (0.45, 0.52) |
|  | 6 Months | 103 | 0.49 (0.45, 0.52) |  | 97 | 0.54 (0.50, 0.58) |
|  | 12 Months | 71 | 0.52 (0.47, 0.57) |  | 68 | 0.56 (0.51, 0.62) |
|  |  |  |  |  |  |  |
| EQ-5D-Y Utility | Baseline | 123 | 0.33 (0.27, 0.40) |  | 117 | 0.32 (0.26, 0.39) |
|  | 6 Months | 104 | 0.36 (0.28, 0.44) |  | 98 | 0.38 (0.30, 0.47) |
|  | 12 Months | 71 | 0.44 (0.36, 0.53) |  | 69 | 0.40 (0.29, 0.50) |

a. VAS scores have been transformed from a 0-100 to 0-1 scale.

**Supplementary Table 9:** Cost-Effectiveness (based on multiple imputation) using EQ-5D-3L values, participants as allocated to Graded Exercise Therapy (GET) or Activity Management (AM)

|  | **GET (n=123)**  Mean (95% CI) | **AM (n=117**^a^**)**  Mean (95% CI) | **Adjusted difference**  **(95% CI)** |
| --- | --- | --- | --- |
| EQ-5D-3L QALY | 0.38  (0.35, 0.42) | 0.37  (0.33, 0.41) | 0.02  (-0.04, 0.07) |
|  |  |  |  |
| Cost (£) | 1735.43  (1407.56, 2063.30) | 1724.26  (1394.00, 2054.52) | 11.17  (-464.92, 487.27) |
|  |  |  |  |
| INMB at £20,000 (£) |  |  | 317.09  (-1010.66, 1644.85) |
| CE % |  |  | 68.20% |
|  |  |  |  |
| INMB at £30,000 (£) |  |  | 484.09  (-1389.78, 2357.96) |
| CE % |  |  | 69.55% |

a. One participant dropped out immediately after random allocation and provided no data

**Supplementary Figure 1: Primary outcome scatter plots (6 month physical function by baseline physical function) in each treatment group.**

**
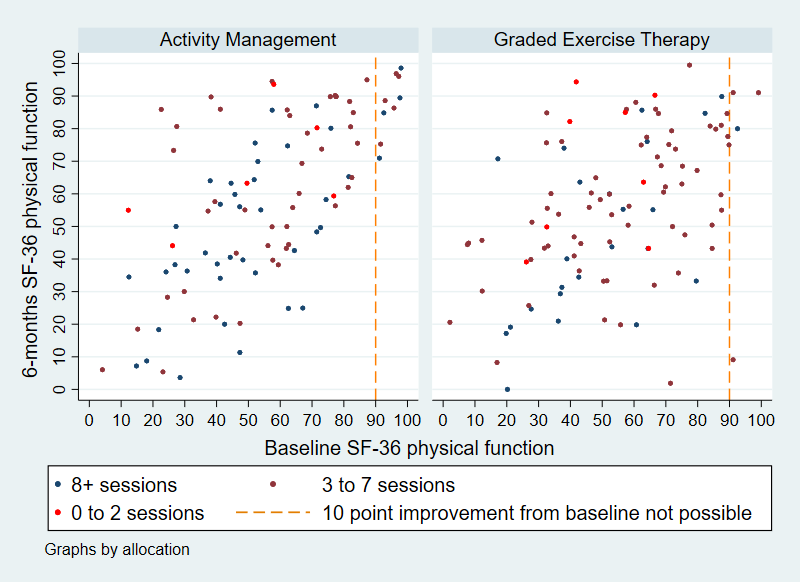
**

**Foot note:** The correlation between baseline and six-month SF-36-PFS was 0.56 overall (0.66 AM and 0.46 GET).

**Supplementary Figure 2.** Number of treatment sessions

| **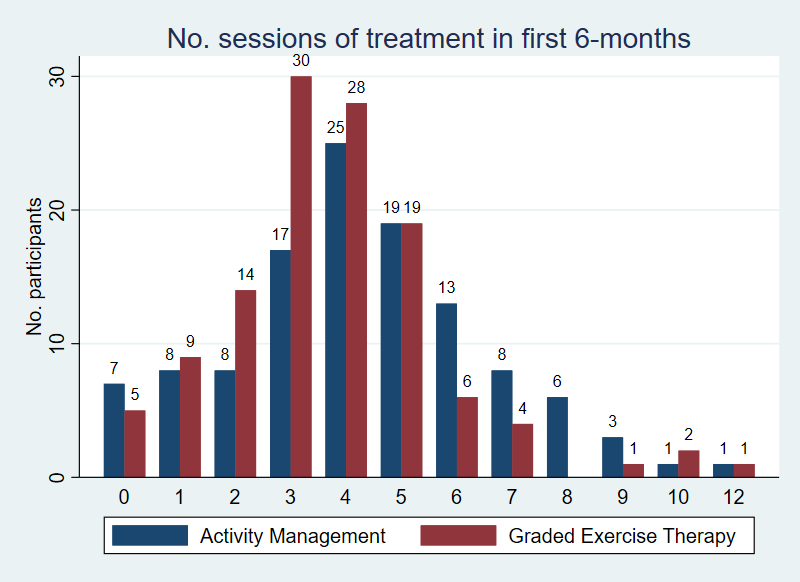** | **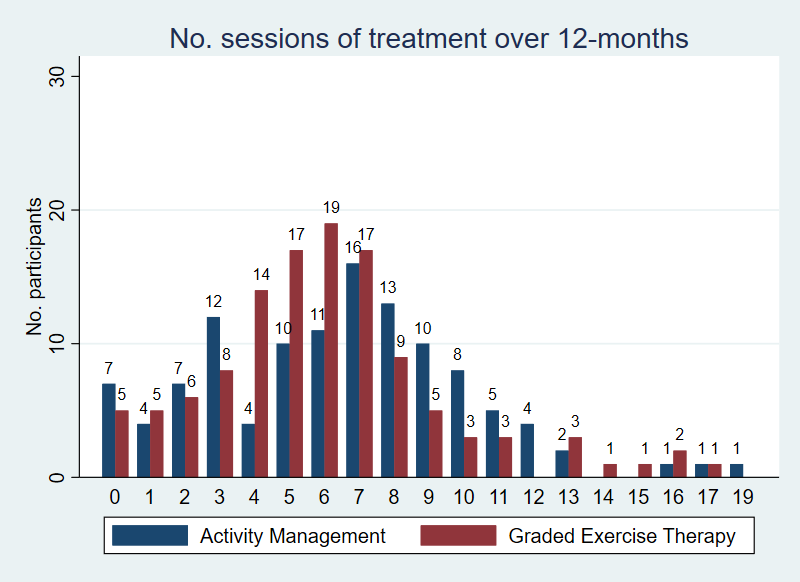** |
| --- | --- |

Supplementary Figure 3: Cost-Effectiveness Acceptability Curve at 12-months
