# Supplementary material for: Graded exercise therapy compared to activity management for paediatric chronic fatigue syndrome/myalgic encephalomyelitis: pragmatic randomized controlled trial
Source: Eur J Pediatr. 2024 Mar 2;183(5):2343–51. doi: 10.1007/s00431-024-05458-x (PMC11035451; doi:10.1007/s00431-024-05458-x)
Supplement: Supplementary file 2 — Supplementary file2 (DOCX 21 KB) [file 431_2024_5458_MOESM2_ESM.docx]

**Appendix 1: Summary of HRA Amendments**

A favourable ethical opinion was given on 03/ 07/2015 (reference 15/SW/0124) by NRES Committee South West - Frenchay. Five favourable opinions were provided for substantial amendments to the study documents and protocol (29/01/2016, 31/03/2016, 27/07/2016, 06/03/17, 25/05/2017). Table 1 below provides a summary of the substantial amendments.

**Table 1: Summary of amendments**

| Amendment number, date of favourable opinion | Summary of substantial amendments |
| --- | --- |
| 1, 29/01/16 | **Amendments to the consent to study forms for parents of participants age 16-17:**  Participants aged 16-17 did not need parents to consent on their behalf, however parents were being asked to complete parental questionnaires and needed to provide consent to this research procedure. A consent form for parents/carers of young people aged 16-17 years was added.  **Amendments to the consent to study forms:**  We intended to use data collected during the feasibility study for the full-scale trial. This was stated in the protocol and the participant information sheets. The consent to study form was updated to reflect this.  **Amendments to the protocol:**  We amended the protocol to allow qualitative interviews via Skype.  **Amendment to the consent to contact, and consent to discussion forms:**  We changed the wording and added a field. |
| 2, 31/03/16 | **Amendments to the protocol:**  Amendments were made to protocol in response to reviewers’ comments upon the publication of the MAGENTA feasibility protocol. This included changes to the background information, aims and objective, the methods, adding stop-go criteria and safety outcomes. |
| 3, 27/07/16 | **Amendments to the Health Economics questionnaires:**  We amended the forms with the aim of improving response rates. We developed a second version of the baseline, 6-month and 12-month questionnaires. The new forms continued to capture our primary outcomes.  **Amendment to document the close down of the Cambridge Site:**  We were unable to recruit from the Cambridge site and documented the sites closure in the protocol.  **Amendments to the protocol:**  We changed wording in the protocol to provided further detail and clarification in the background and methods sections. |
| 4, 06/03/17 | **Amendment to document the transition from feasibility phase to the full trial phase.**  We amended the protocol to reflect the procedures for the full trial (including the sections background, aims & objectives, sample size, analysis, integrated qualitative research). We also amended the participant information sheets and consent forms. We updated study documents with the correct address of the clinical team.  **Amendment to introduce a new mood disorder diagnostic assessment.**  The clinical service were concerned that they were not identifying significant mood disorders at assessment. The clinical service and research team had introduced a new diagnostic assessment. We made changes to the protocol and patient information sheet to detail the procedure for this new assessment.  **Amendment to the participant information sheet to improve equipoise of information delivery.**  To improve equipoise, we changed the wording "group 1" to "activity management" and "group 2" to "graded exercise therapy". |
| 5, 25/05/17 | **Amendment to document the close down of the Newcastle Site:**  We were unable to recruit from the Newcastle site and documented the sites closure in the protocol.  **Amendments to the Health Economics questionnaires:**  With the aim of improving response rates to these questionnaires, we reduced the number of questions, changed the email which accompanies the questionnaire, and introduced a procedure to collect these data over the telephone.  **Amendments to the outcome measure questionnaires:**  If participants did not complete their questionnaire, they were sent two reminder emails, the first with a link to the full version of the questionnaire, the second with a link to a reduced version. We amended this procedure so both reminders included a link to the reduced version of the questionnaire. We also amended the timings of the follow-up reminders. We also shortened the reduced questionnaire so that it contained three measures only (SF-36-PFS, the Chalder Fatigue Scale, the EQ-5D-Y). |
