# Supplementary material for: Graded exercise therapy compared to activity management for paediatric chronic fatigue syndrome/myalgic encephalomyelitis: pragmatic randomized controlled trial
Source: Eur J Pediatr. 2024 Mar 2;183(5):2343–51. doi: 10.1007/s00431-024-05458-x (PMC11035451; doi:10.1007/s00431-024-05458-x)
Supplement: Supplementary file 3 — Supplementary file3 (DOCX 27 KB) [file 431_2024_5458_MOESM3_ESM.docx]

| Activity Management Intervention Monitoring: Intervention |
| --- |

Research ID:

Date of Appointment: 6 Month Due:

Session Number: *Please use 6 month form if applicable

| **Mandatory Tick if discussed Reason if not discussed** | | |
| --- | --- | --- |
| Has the participants managed the baseline for 1-2 weeks? Y / N if yes: | | |
| Increase this baseline by 10-20% each week. |  |  |
| Participants will continue to increase activity until they are able to do at least 8 hours of cognitive activity a day. |  |  |
| All participants: | | |
| The different types of cognitive activity (high concentration and low concentration) which will vary according to age  Cognitive activities include time at school or doing school work, reading, some craft/hobbies, socialising and screen time (phone, laptop, TV, computer, other devices) |  |  |
| Advise participants to record the total number of minutes spent each day doing high-energy cognitive activities using paper diaries/ “ActiveME” app. Recording activity is used to help participants understand whether they are doing the same each day or varying their activity and whether the baseline has been set at the correct level. |  |  |
| Therapists will discuss problems encountered by participants and provide possible solutions. Managing setbacks will be discussed (how much to reduce school and other cognitive activity and for how long). |  |  |

| **Prohibited Tick if discussed Reason if discussed** | | |
| --- | --- | --- |
| Discussion about number of steps, minutes of exercise etc. |  |  |
| Aerobic, versus non aerobic activity |  |  |
| No discussion about increasing physical activity (only discussion about increasing overall activity) |  |  |
| No advice on exercises or using a strengthening programme |  |  |

| **Flexible Tick if discussed** | | |
| --- | --- | --- |
| Advice on PE in school (no PE, half a lesson, full lesson) |  |  |
| Attendance at sporting events (do not attend, attend limited period of time) |  |  |
| Children can record physical activity within the total cognitive activity but are not required to do so |  |  |

| Referred to CBT Y / N |
| --- |
| Has an Adverse even been reported Y / N (if yes, please find adverse event form at the back of this  Pack and please notify research team) |

**Activity Management Intervention Overview**

Activity management aims **to convert a “boom-bust” pattern of to a baseline with the same daily amount**. For children/teenagers with CFS/ME these are almost entirely cognitive activities: school, school work, reading, socialising, and screen time (phone, laptop, TV, games). Those allocated to this arm will receive advice about the total amount of daily activity, including physical activity, but will not receive specific advice about their use of exercise, increasing exercise or timed physical exercise.

Therapists treating children in both arms will be encouraged to offer routine advice about sleep, medication use and symptom control.

Children, their parents/carers and the clinician providing treatment will choose the number of follow up sessions (between 8 & 12) and the frequency of appointments (every 2-6 weeks) within a maximum length of treatment of one year. We will collect the number, frequency and length of follow up sessions for each participant as well as data on heart rate monitor use.

Participants who develop anxiety or depression that require treatment during the trial follow up period will be offered up to 12 sessions of CBT delivered as individual sessions every 2 weeks by a CFS/ME specialist clinical psychologist.

If participants, their parents/carers or therapists feel they would benefit from the alternative treatment arm, they will be able to cross-over after 6 months (the primary outcome) and this will be recorded. Participants will be strongly encouraged not to cross over before 6 months however they are allowed to withdraw from either treatment or the trial at any time. Therefore, if participants want to cross over before three months, this decision will be recorded and they will be encouraged to continue to provide outcome data.

| GET Intervention Monitoring: Ongoing Intervention |
| --- |

Research ID:

Date of Appointment: 6 Month Due:

Session Number: *Please use 6 month form if applicable

| **Mandatory Tick if discussed Reason if not discussed** | | |
| --- | --- | --- |
| Has initial exercise target been achieved for everyday for 1-2weeks? Y / N if yes: | | |
| Child advised to increase exercise slowly by 10-20% a week |  |  |
| Is the child doing 30 minutes of gentle exercise each day Y / N if yes: | | |
| Child advised to increase exercise intensity such that participants start doing aerobic exercise |  |  |
| The aerobic component will then be slowly increased as the total amount of exercise is increased |  |  |
| Participants will be encouraged to continue to increase exercise to achieve Department of Health recommended levels of 60 minutes a day of a mixture of moderate/vigorous intensity aerobic with muscle strengthening activities on three days/week. |  |  |
|  | | |
| For all participants: | | |
| Child advised to continue to time and record their exercise |  |  |
| Diary reviewed to help children ensure their exercise is the same every day |  |  |
| Did the child use the diary? Y / N | | |
| Child advised to continue to monitor their heart rate using a heart rate monitor. Target set. |  |  |
| Has child been using heart rate monitor Y / N | | |
| Managing setbacks discussed prior to discharge in the context of physical exercise (how much this should be reduced and when they should start to do exercise again). |  |  |

| **Prohibited Tick if discussed Reason if discussed** | | |
| --- | --- | --- |
| Advice on cognitive activity |  |  |
| Discussion about the different types of cognitive activities |  |  |
| Instructions to record the cognitive activities |  |  |

**PTO**

| **Flexible Tick if discussed** | | |
| --- | --- | --- |
| Assessment of range of movement. |  |  |
| Advice on length of time at school (full /half days, one lesson a day) and support increasing time at school. |  |  |
| Advice over exams |  |  |
| Children can be shown how to do stretches |  |  |
| They can also be offered a strengthening programme if this is one of their goals |  |  |

| Has an Adverse even been reported Y / N (if yes, please find adverse event form at the back of this  Pack and please notify research team) |
| --- |
| Referred to CBT Y / N |

**GET Intervention Overview**

Children will be offered **advice that is focussed on exercise** with **detailed assessment of current physical activity, advice about exercise** and **a programme including timed daily exercise**. Children will be asked to **record the amount of exercise** and taught to **use a heart rate monitor** with target heart rates to avoid overexertion. They will be able to choose whether they want text reminders to do exercise.

The exercise programme will be negotiated and agreed together at each appointment between the therapist, child and parent/carer.

Therapists treating children in both arms will be encouraged to **offer routine advice about sleep, medication use and symptom control**.

Children, their parents/carers and the clinician providing treatment will choose the number of follow up sessions (between 8 & 12) and the frequency of appointments (every 2-6 weeks) within a maximum length of treatment of one year. We will collect the number, frequency and length of follow up sessions for each participant as well as data on heart rate monitor use.

Participants who develop anxiety or depression that require treatment during the trial follow up period will be offered up to 12 sessions of CBT delivered as individual sessions every 2 weeks by a CFS/ME specialist clinical psychologist.

If participants, their parents/carers or therapists feel they would benefit from the alternative treatment arm, they will be able to cross-over after 6 months (the primary outcome) and this will be recorded. Participants will be strongly encouraged not to cross over before 6 months however they are allowed to withdraw from either treatment or the trial at any time. Therefore, if participants want to cross over before three months, this decision will be recorded and they will be encouraged to continue to provide outcome data.
