# Supplementary material for: Graded exercise therapy compared to activity management for paediatric chronic fatigue syndrome/myalgic encephalomyelitis: pragmatic randomized controlled trial
Source: Eur J Pediatr. 2024 Mar 2;183(5):2343–51. doi: 10.1007/s00431-024-05458-x (PMC11035451; doi:10.1007/s00431-024-05458-x)
Supplement: Supplementary file 4 — Supplementary file4 (DOCX 18 KB) [file 431_2024_5458_MOESM4_ESM.docx]

**Appendix 3** Additional information on Methods

**Accelerometers:** At each time-point, participants were asked to wear the accelerometer for seven days during all waking hours. The minimum requirement for a valid data return was three days (two weekdays and one weekend) for 500 minutes a day. Stamped-addressed envelopes were included to return the accelerometers. Physical activity patterns at baseline and further details on protocols have been published.^33^ The accelerometer data (counts per minute) were processed using Kinesoft (v3.3.75; Kinesoft, Saskatchewan, Canada) to identify mean daily minutes of sedentary, light- and moderate-to-vigorous-intensity physical activity using established accelerometer cut-off points and protocols.^34^

**Imputation model:** The imputation model included age, gender, EQ-5D-Y VAS and preference scores, SF-36 PFS, and baseline values of the SCAS, HADS and Chalder fatigue scales.^39^
