# Supplementary material for: Graded exercise therapy compared to activity management for paediatric chronic fatigue syndrome/myalgic encephalomyelitis: pragmatic randomized controlled trial
Source: Eur J Pediatr. 2024 Mar 2;183(5):2343–51. doi: 10.1007/s00431-024-05458-x (PMC11035451; doi:10.1007/s00431-024-05458-x)
Supplement: Supplementary file 5 — Supplementary file5 (DOCX 88 KB) [file 431_2024_5458_MOESM5_ESM.docx]

The MAGENTA protocol: A randomised controlled trial investigating the effectiveness and cost effectiveness of graded exercise therapy compared to activity management for paediatric CFS/ME

Principal Investigator: Esther Crawley

Members of the Trial Management Group: Chris Metcalfe, Nicola Mills, Will Hollingworth, Russ Jago, Daisy Gaunt

Researchers: Lucy Beasant and Amberly Brigden

Contents

[BACKGROUND 2](#_Toc461794820)

[METHOD 4](#_Toc461794821)

[Recruitment: 4](#_Toc461794822)

[Inclusion/exclusion criteria: 4](#_Toc461794823)

[Arrangements for allocating participants to trial groups: 5](#_Toc461794824)

[INTERVENTIONS 6](#_Toc461794825)

[Activity management 6](#_Toc461794826)

[Graded Exercise Therapy (GET) 6](#_Toc461794827)

[Treatment delivery: 7](#_Toc461794828)

[SAMPLE SIZE 9](#_Toc461794829)

[OUTCOME MEASURES 9](#_Toc461794830)

[Secondary outcome measures 9](#_Toc461794831)

[Measurements for Health Economic evaluation 10](#_Toc461794832)

[Follow up: 10](#_Toc461794833)

[Safety outcomes: 10](#_Toc461794834)

[ANALYSIS 12](#_Toc461794835)

[Primary Outcome 12](#_Toc461794836)

[Secondary Outcomes 12](#_Toc461794837)

[Accelerometer Data 12](#_Toc461794838)

[Health Economics 12](#_Toc461794839)

[Co-morbid Mood Disorders 13](#_Toc461794840)

[Safety Analysis 13](#_Toc461794841)

[INTERGRATED QUALITATIVE RESEARCH 14](#_Toc461794842)

[Ethics 15](#_Toc461794843)

[Storage of data and data protection 15](#_Toc461794844)

[Withdrawal from the study 15](#_Toc461794845)

[Ethical Issues 15](#_Toc461794846)

[Funding 16](#_Toc461794847)

# BACKGROUND

Chronic fatigue syndrome or myalgic encephalomyelitis (CFS/ME) in children is relatively common affecting between 0.1-2% of secondary school children[^1-4^](#_ENREF_1). CFS/ME is defined as “generalised fatigue, causing disruption of daily life, persisting after routine tests and investigations have failed to identify an obvious underlying ‘cause’[^5^](#_ENREF_5) [^6^](#_ENREF_6). National Institute of Health & Clinical Excellence (NICE) guidelines recommend a minimum 3 months duration of fatigue before making a diagnosis in children[^5^](#_ENREF_5).

NICE recommends that children and adolescents with CFS/ME are offered either Cognitive Behavioural Therapy (CBT), Graded Exercise Therapy (GET) or Activity Management[^5^](#_ENREF_5). GET stabilises physical activity levels, before gradually increasing at a manageable rate. Activity Management establishes a baseline for all activity (mainly cognitive, such as school and homework, in children and adolescents) which is then increased[^5^](#_ENREF_5) [^7^](#_ENREF_7). There is good evidence for the effectiveness of CBT in children with CFS/ME [^8-10^](#_ENREF_8), however there is little evidence for the effectiveness of GET in children and adolescents although GET is moderately effective in adults [^11^](#_ENREF_11). There is also limited evidence of the acceptability of GET for children and adolescents with CFS/ME or on the best method for delivering these interventions in terms of intensity (frequency of sessions) and length of intervention (number of sessions and length of time for follow up).

An internal feasibility study has shown that a trial comparing GET and activity management is feasible and that the interventions are acceptable to children and adolescents with CFS/ME and their parents. Between September 2015 and August 2016 our lead site recruited 82 participants (50% of those eligible). Retention rates are good as more than 90% of children and adolescents available for follow up had completed the primary outcome data at 6 months (July 2016). Integrated qualitative methods have shown that GET is acceptable as an intervention for children and their parents.

Co-morbid anxiety and depression affect more than 30% of children with CFS/ME [^12^](#_ENREF_12) [^13^](#_ENREF_13) however, little is known about how to screen for mood disorders in this patient group. The symptoms experienced by children with CFS/ME are similar to those with depression, making screening more complicated. Trials in adults suggest prognosis is worse in those with co-morbid depression [^14-17^](#_ENREF_14). During the feasibility study, anecdotal reports suggest the Hospital Anxiety and Depression Scale is not a sufficiently robust screening tool for children with CFS/ME and significant mood disorders. We are therefore introducing the “gold standard” Kiddie Schedule for Affective Disorders and Schizophrenia (KSADS) for children recruited to MAGENTA to improve the diagnosis of co-morbid mood and anxiety disorders in this patient group.

In this study we will investigate the effectiveness and cost-effectiveness of GET compared to Activity management as part of a multicentre randomised controlled trial. Integrated qualitative methods will be used to explore issues with recruitment, retention and the delivery of the intervention.

## Aims and objectives

The overall aim of this study is to investigate the effectiveness and cost-effectiveness of Graded Exercise Therapy compared to Activity Management for the treatment of CFS/ME in children and adolescents.

*Objective 1. Estimate the effectiveness of Graded Exercise Therapy compared to Activity Management for paediatric CFS/ME.*

*Objective 2. Estimate the cost effectiveness of Graded Exercise Therapy and Activity Management*

# METHOD

## Recruitment:

Paediatricians and GPs refer children and adolescents with probable CFS/ME to the Bath and Newcastle specialist CFS/ME services. Eligible children and adolescents and their families will be identified by the clinician conducting the initial assessment in the CFS/ME specialist service. The clinician will briefly describe the study and give interested families a study pack which contains: age appropriate patient information sheets for the young person and their parents as well as the relevant consent or assent forms. The clinician will provide information about the study and obtain written assent/consent for a member of the research team to talk to the young person and parent/carers about the study and for this discussion to be recorded.

The recruiting researcher will be based in the hospital and potential participants can either meet with the recruiter in the hospital on the day of the initial assessment (if the recruiter is available and the family have time), discuss the study on the phone, meet face to face at clinic or meet over skype within seven days of the initial assessment. At the start of the recruitment discussion (face to face, phone call or via skype), the recruiter will confirm consent/assent for the discussion and check that the parent/young person continues to be happy to have the discussion recorded. Once the recording has started, the recruiter will confirm that consent/assent has been given for the discussion to be recorded before discussing the MAGENTA trial, the study design, interventions, participant burden, potential risks and benefits of taking part.

Young people and parents/carers who wish to take part in the study can either sign the written study consent/assent forms when they meet the recruiter or post the form to the recruiter later. Those who talk to the recruiter on the phone can either sign the study consent/assent forms and post them back to the recruiter or sign the web based consent form provided through the University of Bristol’s data capture system (Research Electronic Data Capture (REDCap, http://project-redcap.org/).

Once the recruiter has received the signed consent/assent form, for young people age 11+, they will pass the details of the participant to a Clinical Psychologist, who will contact the family within seven days to arrange to do the research assessments for this study (KSADS interview).The results of the KSADS will be given to the clinician responsible (with permission from the participant and family). The clinician will discuss the results at follow up.

## Inclusion/exclusion criteria:

Children and adolescents will be eligible for inclusion if they are given a diagnosis of CFS/ME (made using NICE guidance)^5^ at clinical assessment and aged between 8 and 17 years inclusive.

Children and adolescents will be excluded if they are severely affected by CFS/ME. NICE defines severe CFS/ME as individuals who are unable to do activity for themselves, or carry out minimal daily tasks only, or they have severe cognitive difficulties or depend on wheelchair for mobility[^5^](#_ENREF_5). Patients will not be eligible if they are referred for CBT at their first clinical assessment; or are unable to attend clinical/ Skype sessions. Eligibility assessment will be carried out by the clinician at assessment and checked by the recruiting researcher.

*Screening logs* will include information on every young person assessed in each centre. Screening logs will be maintained by a member of the research team in each location who will collaborate with the lead clinician. Logs will include details on whether the young person was approached to take part in the study. If the young person was not approached the reason will be recorded (e.g. clinician forgot, or not eligible). If the young person is not eligible, the reason will be recorded. The research number will be linked to the patient name and the link will be held separately in the NHS centre.

## Randomisation :

Once the recruiter has received the signed consent/assent form they will use the automated telephone/web randomisation service operated by the Bristol Randomised Trials Collaboration. Allocation to the two treatment arms of either Graded Exercise Therapy (GET) or Activity Management (AM) (allocation ratio 1:1) will use minimisation to facilitate balance between trial arms by age (categories are 8-12, 13-17) and gender, and stratified by centre. We will retain a random component to prevent accurate prediction of allocation. Because of the nature of the intervention, it is not practical to blind either the family or the clinical service to treatment allocation. If allocation is done during the recruitment appointment, families are told the allocation immediately. If the allocation is done later the recruiter will phone the family with their allocation.

After allocation the recruiter will inform the clinical service who will then write to the young person /family with their appointment details. GPs will be told what intervention the young person will receive as part of routine clinical practice.

# INTERVENTIONS

Therapists treating children and adolescents in both arms will be encouraged to offer routine[^5^](#_ENREF_5) advice about sleep, medication use and symptom control at the assessment and follow up appointments in both treatment arms.

Activity management

Activity Management will be delivered by CFS/ME specialists (occupational therapists, physiotherapists, nurses, psychologists). As Activity Management is currently being delivered in both services, therapists will not require further training however therapists will receive guidance on the mandatory, prohibited and flexible components detailed below. Activity management aims to convert a “boom-bust” pattern of activity (lots one day and little the next) to a baseline with the same daily amount. For children/adolescents with CFS/ME these are almost entirely cognitive activities: school, school work, reading, socialising, and screen time (phone, laptop, TV, games). Those allocated to this treatment arm will receive advice about the total amount of daily activity, including physical activity, but will not receive specific advice about their use of exercise, increasing exercise or timed physical exercise.

*Mandatory:* Therapists will discuss the different types of cognitive activity (high concentration and low concentration) which will vary according to age. Participants will be taught how to find their baseline of cognitive activities. Cognitive activities include time at school or doing school work, reading, some craft/hobbies, socialising and screen time (phone, laptop, TV, computer, other devices). The baseline is the median time spent doing cognitive activity and can either be estimated in collaboration with the specialist therapist or calculated after a period of recording activity. Once the baseline is agreed with participants, they will be asked to record the total number of minutes spent each day doing high-energy cognitive activities using paper diaries or our award-winning smartphone app “ActiveME”. Recording activity is used to help participants understand whether they are doing the same each day or varying their activity and whether the baseline has been set at the correct level. When participants have managed the baseline for 1-2 weeks, they will be asked to increase this by 10-20% each week[^5^](#_ENREF_5). Therapists will discuss problems encountered by participants and provide possible solutions.

Managing setbacks will be discussed (how much to reduce school and other cognitive activity and for how long). Participants will continue to increase activity until they are able to do at least 8 hours of cognitive activity a day.

Therapists will complete a tickbox checklist for each session to record which mandatory elements were provided.

*Prohibited:* Discussion about number of steps, minutes of exercise, aerobic, versus non aerobic activity. No clinician initiated discussion about increasing physical activity (only discussion about increasing overall activity) or using a strengthening programme.

*Flexible:* Advice on PE in school (no PE, half a lesson, full lesson). Attendance at sporting events (do not attend, attend limited period of time). Children and young people can record physical activity within the total activity but are not required to do so.

Graded Exercise Therapy (GET)

GET will be delivered by referral to a GET-trained CFS/ME specialist who will receive guidance on the mandatory, prohibited and flexible components detailed below. Children and adolescents will be offered advice that is focused on exercise with detailed assessment of current physical activity, advice about exercise and a programme including timed daily exercise. Young people will be asked to record the amount of exercise. All participants will be taught to take their heart rate to avoid overexertion.

*Mandatory:* Physical assessment, assessment of range and type of exercise used during the week at the first assessment. Tests at assessment and 6 months include: sit to stand, step test, and 1 minute balance test. Exercise targets will be negotiated with the young people and parents/carer. Initial exercise targets (the baseline) will be the median amount of daily exercise done during the week. Once this is achieved every day for one to two weeks, participants will be advised to increase exercise slowly by 10-20% a week. They will be asked to time their exercise to make sure they are completing the same number of minutes of exercise every day and record these minutes of exercise each day using either paper diaries or our smartphone app “ActiveME”. Diaries (paper or using ActiveME) will be reviewed to help children and adolescents ensure their exercise is the same every day. Once children and adolescents are doing 30 minutes of gentle exercise each day, the exercise will increase in intensity such that participants start doing aerobic exercise. The aerobic component will then be slowly increased as the total amount of exercise is increased. The exercise programme will be negotiated and agreed together at each appointment between the therapist, young person and parent/carer.

Participants will be taught how to manually monitor their heart rate to prevent them doing too much exercise. They will be set a target heart rate and asked not to exceed this. If the clinician feels it is appropriate participants will be offered a Fitbit to assist with heart rate monitoring.

Managing setbacks will be discussed prior to discharge in the context of physical exercise (how much this should be reduced and when they should start to do exercise again).

Participants will be encouraged to continue to increase exercise to achieve Department of Health recommended levels of 60 minutes a day of a mixture of moderate/vigorous intensity aerobic with muscle strengthening activities on three days/week.

Therapists will complete a tickbox checklist for each session to record which mandatory elements were provided.

*Prohibited:* Advice on cognitive activity, discussion about the different types of cognitive activities. Instructions to record the cognitive activities.

*Flexible:* Assessment of range of movement. Advice on length of time at school (full days, half days, one lesson a day), support increasing time at school. Advice over exams. Participants can be shown how to do stretches. They can also be offered a strengthening programme if this is one of their goals.

Treatment delivery

By default follow up sessions will be scheduled every 4 weeks – although therapists will have the discretion to increase (up to 2 weeks) or decrease (down to 6 weeks) the frequency throughout the course of treatment. We anticipate that patients will be offered up to 12 treatment sessions over the initial 26 week period. However, therapists can discontinue treatment earlier or extend it further if indicated. Therapist may offer some appointments via skype in line with standard clinical practice.

Participants who develop anxiety or depression as part of routine care, that requires treatment during the trial follow up period will be offered up to 12 sessions of CBT delivered as individual sessions every 2 weeks by a CFS/ME specialist psychologist.

At recruitment we will emphasise to participants the importance of attending all therapy sessions to give the allocated therapy (AM or GET) the best opportunity to have an effect. Participants can withdraw from either treatment or the trial at any time. At the first clinical appointment after 6 months (when the primary outcome is collected) participants, in discussion with parent/carers and therapists, will be offered the opportunity to cross-over and receive the other therapy if they wish. However, at the request of participants or parents/carers, cross-over will be allowed before 6 months. All decisions to cross-over will be recorded and participants will be encouraged to continue to provide outcome data for the trial.

We will record the number of booked treatment sessions where participants did not arrive or where there was a late cancellation (within 24 hours). We will assume that those who did not attend (or cancelled within 24 hours) three or more consecutive appointments or 50% of appointments did not find the interventions acceptable.

# SAMPLE SIZE

The Minimal Clinically Important Difference (MCID) for the SF-36-PFS is 10 points [^18^](#_ENREF_18) [^19^](#_ENREF_19) which is equal to 0.4SD calculated using the mean SF-36-PFS of 49.8 with SD 24.8 in children with CFS/ME at assessment by the Bath Specialist CFS/ME service. For 80% power at 5% alpha, data on the primary outcome in 200 children is needed. Assuming a loss to follow up of 10%, we need to recruit 222 children into the trial. We will use the outcome data in 80 participants already recruited in the feasibility trial. This means we need to recruit an additional 142 children over the next 18 months which is consistent with our current recruitment rate.

# OUTCOME MEASURES

Baseline data is routinely collected at the first clinical assessment prior to recruitment. Outcomes are collected at 6 months and 12 months post randomisation.

## Primary outcome measure

The primary outcome is SF36 physical function sub scale collected at 6 months after randomisation.

Table 1: Data routinely collected at assessment. Questionnaire data also collected at 6 & 12 months.

| Assessment data | Questionnaires |
| --- | --- |
| Age | Chalder fatigue[^20^](#_ENREF_20) |
| Sex | Physical function (SF 36)[^21^](#_ENREF_21) |
| Ethnicity (drop down list)  School attendance  % possible school | Hospital Anxiety Depression Scale[^22^](#_ENREF_22)  Spence Children’s Anxiety Scale[^23^](#_ENREF_23) |
| Symptoms List  CDC & NICE criteria  Months of illness  Co-morbid conditions  Kiddie Schedule for Affective Disorders and Schizophrenia (within a week of consenting to participate in MAGENTA) | Pain visual analogue scale  Quality of life (EQ-5D-Y) Clinical Global Impressions scale*  Productivity**  Healthcare, education and expenses** |

*Follow up only.

** Completed by parent/carer(s)

Secondary outcome measures

Child self-completed questionnaires measuring: school attendance (percentage of expected sessions); Chalder Fatigue score; pain visual analogue scale, depression and anxiety (Spence Children’s Anxiety Scale (SCAS) and the Hospital Anxiety and Depression Scale (HADS, if they are 12-17 years old), health related quality of life (EQ-5D-Y)) at 6 and 12 months as well as the SF36-PFS at 12 months.

Participants in both trial arms will be asked to wear an accelerometer (GT3X+) to measure physical activity for seven days within one month of randomisation and at 3 and 6 months follow-up. Accelerometers will be posted to participants within 2-4 weeks of their first clinical assessment. Instructions will be included with the accelerometer. Accelerometers are small, match boxed sized devices that measure physical activity. They have been shown to provide reliable indicators of physical activity among children and adults [^24^](#_ENREF_24).

The accelerometer data will be processed to identify mean minutes of sedentary, light and moderate to vigorous intensity physical activity per day using established accelerometer cut-off points and protocols[^25^](#_ENREF_25) [^26^](#_ENREF_26) . The mean accelerometer counts per minute, which provides an indication of the volume of physical activity in which the participant engages, will also be calculated using established methods.

Measurements for Health Economic evaluation

Participants will be asked to complete the EQ-5D-Y at baseline, 6 and 12 months. Up to two parents/carers involved in the young person’s care will be asked to complete inventories at baseline and 6 and 12 months follow up including: an adapted 6 item Work Productivity and Activity Impairment Questionnaire (General Health V2.0 [WPAI:GH])[^27^](#_ENREF_27) and an adapted health resource use questionnaire to measure health service use (e.g. GP or specialist care), educational service (e.g. school counsellor) and travel costs. We have tested the acceptability of these inventories in this participant group. We will extract information from the specialist services medical records to identify additional specialist care (e.g. CBT or Child and Adolescent Mental Health services care) within 12 months of randomisation.

Follow up

We will email participants to complete outcome measures at 6 months and 12 months with a link to the questionnaires on REDCap. If families do not have internet access at home, we will post questionnaires. If outcomes are not completed, an email (or postal) reminder will be sent after one week. If outcomes are still not completed a further email (or postal) reminder will be sent with a link to a reduced number of questions containing: question on school attendance, the Chalder Fatigue Scale the SF-36 PFS, the EQ-5D-Y and the Clinical Global Impressions scale . If this is not completed we will make up to four follow up telephones calls or emails and offer to collect the primary outcome data over the phone.

Safety outcomes

For safety outcomes, we will prospectively collect serious and non-serious adverse events.

Serious adverse events: We will define serious adverse events as: “any adverse event that results in death, is life threatening, requires hospitalisation or prolongation of existing hospitalisation, results in persistent or significant disability or incapacity (<http://www.ct-toolkit.ac.uk/glossary/serious-adverse-event-sae-or-serious-adverse-reaction-sar>). These will be reported by the clinical team/participant or parent/carer to the research team. All SAEs will be reported to the Sponsor the next working day according to the Sponsor’s protocol

Chronic fatigue syndrome or ME (CFS/ME) is by its nature, a fluctuating illness. The description of activity and function in CFS/ME is one of “boom-bust” which usually occurs over several days and sometimes weeks. Exacerbation of symptoms which can be called “payback” or “crashes” or “flares” are to be expected in young people whether or not they are undergoing treatment. Payback, crashes or flares, can mean that a child who was previously mobile becomes bed bound or is unable to go to school. Episodes can last days or occasionally weeks. Treatment is designed to reduce these over time and to help children and parents to learn to manage these setbacks, but the specific risk of flares without treatment, during or post treatment is not known.

Non-serious adverse events: The MAGENTA trial will investigate whether young people randomised to one treatment arm are at a higher risk of unexpected harm in one treatment group compared with the other. We are interested in harm that is not a SAE as defined above. Therefore, in addition to collecting data on SAEs we will also collect the following information:

Clinician defined clinical change or illness reported to the clinician and forwarded on to the study team (clinical-reported serious deterioration in health) during treatment. This will be unexpected or unexplained deterioration in health as defined by the clinician or unexpected or unexplained health outcomes that are not normally seen by CFS/ME specialist clinicians.

We will define a serious deterioration in health as either a decrease in ≥20 in the SF-36-PFS between baseline and 6 or 12 month follow-up, scores of “much” or “very much worse” on the Clinical Global Impression scale at 6 or 12 months or withdrawal from treatment because of feeling worse.

Safety outcomes will be reviewed by the Data and Safety Monitoring Committee (DSMC) and reported to the Trial Steering Committee (TSC).

There has been one safety analysis which took place at 10 months (before the trial transitioned from the feasibility to full study). Subsequent meetings will take place when approximately 50% of patients are recruited. The DSMC will be notified of any emerging concerns and will also receive quarterly SAE reports. These analyses will only investigate safety outcomes and will be conducted by an independent statistician with results provided the data and safety monitoring committee.

The research team will only notify fatal and unexpected non-fatal adverse events to the trial sponsor. Expected adverse events include payback, crashes or flares as described above. Expected adverse events will not be reported to the sponsor.

All adverse events will be recorded in detail on a case record form. At the conclusion of the study and during the safety analyses, all adverse events recorded during the study will be subject to statistical analyses and the analyses and subsequent conclusion will be included in the final study report.

For all unexpected serious adverse events, the subject will be actively followed up, and the investigator (or delegated person) will provide follow-up every five working days after the initial report until the serious adverse event has resolved or a decision for no further follow – up has been taken.

Data on adverse events will be collected for each participant from the point at which they consent to take part in the study until the end of the follow-up period (12 months).

ANALYSES

Analyses and presentation of the trial will be in accordance with CONSORT guidelines. A full statistical analysis plan (SAP) will be developed and agreed with the Trial Management Group, DSMC and Trial Steering Committee prior to any data analyses.

Appropriate descriptive statistics will be used to compare characteristics of invited individuals who did or did not agree to take part and eligible individuals who were or were not randomised. We will examine the balance in participant characteristics between trial arms.

## Primary Outcome

We will compare the mean SF-36-PFS scores (primary outcome) at six months according to randomised allocation among participants with measured outcomes, using multivariable linear regression adjusting for baseline values of the outcome, baseline age and gender. For the primary outcome, we will conduct sensitivity analyses in which we adjust our primary analysis model for prognostic variables for which there is a baseline imbalance between intervention arms. Further sensitivity analyses will use appropriate imputation methods to deal with missing data (if applicable).

## Secondary Outcomes

Similar regression analyses will be conducted for secondary outcomes (linear regression for numerical outcomes and logistic regression for binary outcomes). The secondary outcome of 12-month SF-36-PFS scores will be analysed using the same model as the primary outcome.

## Accelerometer Data

We will use data collected from accelerometers to quantify physical activity levels in both intervention groups and examine whether changes in activity mediate or are associated with outcome. Periods of ≥60 minutes of zero values will be defined as accelerometer “non-wear” time and discarded. Participants will be included in the analysis if they provide ≥3 weekdays of data with at least 500 minutes of data between 6am and 11pm. Mean minutes of weekday, light (LPA) and moderate-to-vigorous physical activity (MVPA) per day will be established for weekdays using the threshold developed by Evenson and colleagues[^25^](#_ENREF_25) , which has been shown to be the most accurate for this age group. The mean number of accelerometer counts per minute (CPM), which provides an indication of the overall volume of activity in which children engage, will be calculated.

Sensitivity analyses will be conducted which will include those participants who provided at least 1 or 2 days (weekday or weekend) of valid accelerometer data with models re-run and results qualitatively compared.

## Health Economics

Our economic evaluation will compare the cost-effectiveness of GET versus Activity Management. We will calculate Quality Adjusted Life Years (QALYs) based on EQ-5D-Y responses at baseline 6 and 12 months. UK value sets for the EQ-5D-Y to calculate utility scores and derive QALYs are currently in development and expected to be available by the end of this RCT. In our primary economic evaluation we will collate the cumulative NHS service use recorded on trial forms, the specialist care notes and reported by parents/carers. We will use national reference costs, where available, to value service use. We will estimate the additional cost per QALY gained of GET versus AM, in line with NICE Guide to the Methods for Technology Appraisal and use net benefit regression or similar approach to adjust for baseline age and gender and estimate uncertainty around our primary estimate and plot a cost-effectiveness acceptability curve. In line with the statistical analyses we may use sensitivity analyses to adjust for other prognostic variables with baseline imbalance and to test how robust our results are to imputation of missing data.

In secondary economic analyses we will examine the wider impact of treatment on parent/carer work, productivity, usual activities, lost wages and other family expenses.

## Co-morbid Mood Disorders

To examine the prevalence of co-morbid depression and anxiety, participants will be defined as having co-morbid depression (or not) and co-morbid anxiety (or not) using the K-SADS, which results from a conclusion about whether an individual meets the DSM-V diagnosis of depression and/or anxiety.

## Safety Analysis

The Data and Safety Monitoring Committee (DSMC) will specify how many independent safety analyses should be conducted and when these should be done. These analyses will only investigate safety outcomes and will be conducted by a statistician with un-blinded results provided to the DSMC. These data will be reviewed by the Trial Management Group and the DSMC, and reported to the Trial Steering Committee.

# INTERGRATED QUALITATIVE RESEARCH

Integrated qualitative methods will be used to explore issues with recruitment, retention and the delivery of the intervention

This research will be flexible in its intensity and comprehensiveness depending on the type of issues that emerge. Sources of difficulties will be fed back to the chief investigator (CI) and trial management group and suggestions made to change aspects of the design, conduct, organisation or training that could then lead on to improvements in how the trial is conducted.

Recruiting staff will receive on-going training sessions when necessary, (e.g. if a new recruiter joins the team). To identify any on-going recruitment difficulties and improve recruitment[^28^](#_ENREF_28) we will continue to audio-record (with consent) all recruitment consultations. The research team will monitor the outcome of the recruitment consultations and analyse the audio-recorded data when necessary, (e.g. if high numbers of patients decline the trial, or one of the trial interventions). If analyses of the audio-recordings suggest that any recruitment difficulties are being caused by the recruitment discussion (for example, that one treatment arm is emphasised over another), training will be offered with the recruiter and a tips guide developed, based on findings from the audio-recorded data. This may include simply providing feedback on the recruitment discussion or may include suggestions on how to make the discussion more balanced in terms of information given on the different interventions.

If the number of eligible patients recruited decreases significantly or if there are differences in the percentage recruited between centres, we may undertake in-depth interviews with members of the clinical and recruitment staff and analyse screening logs to examine problems with patient pathways in the different centres. We may also interview clinicians delivering both interventions to ascertain their views on changes that need to be made to the interventions offered, engagement, compliance and technical problems.

If patients report issues relating to recruitment/randomisation or the acceptability of their allocated intervention (via feedback to recruiters/clinical staff) we will undertake in-depth interviews with parents/carers and their children to understand their views and experiences of the interventions and wider trial processes. This will include provision and acceptability of patient information and reasons for accepting or declining participation. We are particularly interested in understanding barriers to participation and will interview (subject to informed consent) those who choose not to participate in the trial, who drop out of trial follow up or who do not accept treatment allocation at randomisation. Patients will be interviewed for between 20-30 minutes, and parents for up to 1 hour. Interviews will be carried out via telephone, Skype or in the family home.

Intervention sessions will be audio-recorded when a new member of staff joins the clinical team, (for up to two months) with consent to enable us to ensure that the two interventions are truly distinct and delivered in a consistent manner. Intervention sessions will be analysed by the research team and feedback to new staff will be provided by the CI.

Qualitative analyses: Analysis will be ongoing and iterative commencing soon after data collection and will inform further sampling and data collection. Transcripts from audio-recorded consultations and interviews, along with observation notes, will be imported into NVivo and analysed using content and thematic analysis. Individuals exhibiting contrasting attitudes (‘negative cases’) will be studied in detail. The perspectives of the individuals will be paramount, with careful account taken of the context within which the discussion takes place. Data analyses will primarily be undertaken by the qualitative researcher. To check coding reliability, other members of the team will independently analyse a proportion of transcripts and compare findings.

# Ethics

## Storage of data and data protection

Children and young people are allocated a unique seven digit research identification number. This number is assigned to the patient and is used on clinical assessment forms prior to transfer of data so they are anonymised at source. A list of names and corresponding identification numbers are kept separately and securely on a password protected NHS server. This number will be used on screening logs and on all data collected. Personal information will be kept on consent forms which will have contact details. Consent forms will be kept within a locked filing cabinet in a locked office within the University of Bristol.

Data will be entered into REDCap a secure system used by multiple institutions for large multicentre studies. Assessment data will be entered by the research team as this is collected prior to assessment. Participants will be encouraged to provide follow up data using REDCap but will be able to provide data by post if they do not have internet access. Participants are required to log in to the system and have to pass authentication before they can access their own data. There are several authentication methods available. The University of Bristol will use table-based authentication, which utilizes the storage of username/password pairs in a database table. In this system, the password in the database table is encrypted as a one-way hash of the password. Participants will be sent a web link to REDCap which will only allow access to their data. They will create a password which they will use each time they log in. REDCap also has an auto-log out system that will log participants out after 30 minutes if they have stopped using the database.

Audio-recordings will be encrypted, password protected and stored on a secure university server for five years. This is to enable us to check recordings if necessary while reports are being written. Transcripts will be anonymised and secure password protected university server.

## Withdrawal from the study

Participants can withdraw from the study at any time without giving a reason. If a participant wants to withdraw from the study, they will be asked to inform the MAGENTA project manager. We will retain non-identifiable information already collected from participants but will ask whether they want to withdraw from the intervention, further data collection or both.

## Ethical Issues

Graded Exercise Therapy, Cognitive Behavioural Therapy and Activity Management are recommended as a treatments in NICE guidance[^5^](#_ENREF_5), however there is no evidence that Graded Exercise Therapy is effective or cost effective in young people. CFS/ME is different in children/adolescents and adults with different risk factors, course and outcome[^29^](#_ENREF_29). It is therefore not possible to extrapolate the results from adult studies to children/adolescents. A trial in children and adolescents is therefore needed.

At the moment, CBT has the best evidence for treatment efficacy. We have not included CBT as the control arm because clinicians would not be in equipoise in randomising participants to CBT or GET. This is because currently, clinicians recommend CBT at assessment for those who present with CFS/ME and co-morbid mood problems. We have ensured that CBT continues to be a treatment option for young people who develop mood problems after randomisation and can be accessed in addition to either treatment arm. We will analyse the use of CBT as a secondary outcome using hospital records.

Because the participants will be children and adolescents, we have put in place rigorous procedures for informed consent from parents and guardians on behalf of their children. We will also ensure we have informed consent/assent from participating children and young people. In the clinic, the clinician will ask for consent/assent for contact by a recruiter and qualitative researcher. Consent/assent to the study and to randomisation will be obtained by the recruiter after a full explanation of the study when both the young person and the family have had sufficient opportunity to ask questions. Young people and their families will be given as long as they need before giving consent/assent within the confines of the study. We will then obtain further consent/assent prior to each interview to check that young people or their parents continue to be willing to participate. We will also obtain consent/assent prior to recording any interventions from all present.

## Funding

This trial is funded by the NIHR under the terms of Professor Crawley’s Senior Research Fellowship. The Sponsor for this trial will be the Royal United Hospital, Bath.

REFERENCES

1. Nijhof SL, Maijer K, Bleijenberg G, et al. Adolescent chronic fatigue syndrome: prevalence, incidence, and morbidity. Pediatrics 2011;**127**(5):e1169-e75.

2. Crawley E, Hughes R, Northstone K, et al. Chronic Disabling Fatigue at Age 13 and Association With Family Adversity. Pediatrics 2012;**130**(1):E71-E79.

3. Crawley EM, Emond AM, Sterne JA. Unidentified Chronic Fatigue Syndrome/myalgic encephalomyelitis (CFS/ME) is a major cause of school absence: surveillance outcomes from school-based clinics. BMJ Open 2011;**1**(2):e000252.

4. Chalder T, Goodman R, Wessely S, et al. Epidemiology of chronic fatigue syndrome and self reported myalgic encephalomyelitis in 5-15 year olds: cross sectional study. BMJ 2003;**327**(7416):654-5.

5. NICE. Chronic fatigue syndrome/myalgic encephalomyelitis (or encephalopathy): Diagnosis and management of CFS/ME in adults and children (NICE guidelines CG53). London, 2007.

6. Royal College of Paediatrics and Child Health. Evidence Based Guideline for the Management of CFS/ME (Chronic Fatigue Syndrome/Myalgic Encephalopathy) in Children and Young People. London, 2004.

7. White PD, Sharpe MC, Chalder T, et al. Protocol for the PACE trial: a randomised controlled trial of adaptive pacing, cognitive behaviour therapy, and graded exercise, as supplements to standardised specialist medical care versus standardised specialist medical care alone for patients with the chronic fatigue syndrome/myalgic encephalomyelitis or encephalopathy. BMC Neurol 2007;**7**:6.

8. Stulemeijer M, de Jong LW, Fiselier TJ, et al. Cognitive behaviour therapy for adolescents with chronic fatigue syndrome: randomised controlled trial. BMJ 2005;**330**(7481):14.

9. Al-Haggar M S, Al-Naggar Z A, Abdel-Salam M A. Biofeedback and cognitive behavioural therapy for Egypian adolescents suffering from chronic fatigue syndrome. . J Paediatric Neurol 2006;**4**:8.

10. Nijhof SL, Bleijenberg G, Uiterwaal CS, et al. Effectiveness of internet-based cognitive behavioural treatment for adolescents with chronic fatigue syndrome (FITNET): a randomised controlled trial. Lancet 2012;**379**(9824):1412-18.

11. White PD, Goldsmith KA, Johnson AL, et al. Comparison of adaptive pacing therapy, cognitive behaviour therapy, graded exercise therapy, and specialist medical care for chronic fatigue syndrome (PACE): a randomised trial. Lancet 2011;**377**(9768):823-36.

12. Bould H, Collin SM, Lewis G, et al. Depression in paediatric chronic fatigue syndrome. Arch Dis Child 2013;**98**(6):425-8.

13. Crawley E, Hunt L, Stallard P. Anxiety in children with CFS/ME. Eur Child Adolesc Psychiatry 2009;**18**(11):683-9.

14. Kempke S, Goossens L, Luyten P, et al. Predictors of outcome in a multi-component treatment program for chronic fatigue syndrome. J Affect Disord 2010;**126**(1-2):174-9.

15. Wearden AJ, Dunn G, Dowrick C, et al. Depressive symptoms and pragmatic rehabilitation for chronic fatigue syndrome. Br J Psychiatry 2012;**201**:227-32.

16. Darbishire L, Seed P, Ridsdale L. Predictors of outcome following treatment for chronic fatigue. Br J Psychiatry 2005;**186**:350-1.

17. Flo E, Chalder T. Prevalence and predictors of recovery from chronic fatigue syndrome in a routine clinical practice. Behav Res Ther 2014;**63**:1-8.

18. Wyrwich KW, Fihn SD, Tierney WM, et al. Clinically important changes in health-related quality of life for patients with chronic obstructive pulmonary disease: an expert consensus panel report. J Gen Intern Med 2003;**18**(3):196-202.

19. Wyrwich KW, Spertus JA, Kroenke K, et al. Clinically important differences in health status for patients with heart disease: an expert consensus panel report. Am Heart J 2004;**147**(4):615-22.

20. Chalder T, Berelowitz G, Pawlikowska T, et al. Development of a fatigue scale. J Psychosom Res 1993;**37**(2):147-53.

21. Ware JE, Sherbourne CD. The MOS 36-item short-form health survey (SF-36). I. Conceptual framework and item selection. MedCare 1992;**30**(6):473-83.

22. White D, Leach C, Sims R, et al. Validation of the Hospital Anxiety and Depression Scale for use with adolescents. BrJPsychiatry 1999;**175**:452-54.

23. Spence SH, Barrett PM, Turner CM. Psychometric properties of the Spence Children's Anxiety Scale with young adolescents. JAnxietyDisord 2003;**17**(6):605-25.

24. Welk GJ, Schaben JA, Morrow JR, Jr. Reliability of accelerometry-based activity monitors: a generalizability study. Med Sci Sports Exerc 2004;**36**(9):1637-45.

25. Evenson KR, Catellier DJ, Gill K, et al. Calibration of two objective measures of physical activity for children. J Sports Sci 2008;**26**(14):1557-65.

26. Jago R, Edwards MJ, Sebire SJ, et al. Effect and cost of an after-school dance programme on the physical activity of 11-12 year old girls: The Bristol Girls Dance Project, a school-based cluster randomised controlled trial. Int J Behav Nutr Phys Act 2015;**12**:128.

27. Reilly MC, Zbrozek AS, Dukes EM. The validity and reproducibility of a work productivity and activity impairment instrument. Pharmacoeconomics 1993;**4**(5):353-65.

28. Donovan JL, Lane JA, Peters TJ, et al. Development of a complex intervention improved randomization and informed consent in a randomized controlled trial. JClin Epidemiol 2009;**62**(1):29-36.

29. Crawley E. The epidemiology of chronic fatigue syndrome/myalgic encephalitis in children. Arch Dis Child 2013.
