# Supplementary material for: Graded exercise therapy compared to activity management for paediatric chronic fatigue syndrome/myalgic encephalomyelitis: pragmatic randomized controlled trial
Source: Eur J Pediatr. 2024 Mar 2;183(5):2343–51. doi: 10.1007/s00431-024-05458-x (PMC11035451; doi:10.1007/s00431-024-05458-x)
Supplement: Supplementary file 6 — Supplementary file6 (PDF 1770 KB) [file 431_2024_5458_MOESM6_ESM.pdf]

Bristol Randomised Trials Collaboration

MAGENTA

Managed Activity Graded Exercise in Teenagers and Pre-adolescents

Statistical and Health Economics Analysis Plan

Study Statistician: Daisy Gaunt

Study Health Economist: Shaun Harris

	NAME	TITLE	SIGNATURE	DATE
**Senior Statistician**	Chris Metcalfe	Professor of Medical Statistics	[illegible]	5/11/2019
**Senior Health Economist**	William Hollingworth	Professor of Health Economics	[illegible]	8/11/19
**Study CI**	Esther Crawley	Professor of Child Health	[illegible]	14/11/19.

Table of Contents

Abbreviations 4
1. INTRODUCTION & PURPOSE 5
2. SYNOPSIS OF STUDY DESIGN AND PROCEDURES 6
2.1 Trial objectives and aims 6
2.1.1 Primary objective 6
2.1.2 Secondary (economic) objective 6
2.2 Trial design and configuration 6
2.3 Trial centres 6
2.4 Eligibility criteria 6
2.4.1 Inclusion criteria 6
2.4.2 Exclusion criteria 6
2.5 Description of interventions 7
2.6 Randomisation procedures 7
2.7 Blinding and breaking of blind 8
2.8 Trial committees 8
2.9 Outcome measures 8
2.9.1 Primary outcome 8
2.9.2 Secondary outcomes 8
2.10 Economic data measurement and valuation 9
2.11 Sample size and justification 10
2.12 Interim analysis 10
3. GENERAL ANALYSIS CONSIDERATIONS 11
3.1 Analysis populations 11
3.2 Derived variables 11
3.3 Procedures for missing outcome data 11
3.4 Outliers 11
3.5 Visit windows 12
3.6 Software 12
4. DESCRIPTION OF PARTICIPANT CHARACTERISTICS 13
4.1 Disposition 13
4.2 Baseline characteristics 13

5. ASSESSMENT OF STUDY QUALITY 14
5.1 Eligibility checks 14
5.2 Data validation 14
5.3 Intervention fidelity and adherence 14
5.4 Protocol deviations 14
5.5 Changes made to the planned statistical analyses 14
6. ANALYSIS OF EFFECTIVENESS 15
6.1 Primary analysis 15
6.2 Secondary analyses 15
6.3 Sensitivity analysis 16
6.4 Sub-group analyses 16
7. HEALTH ECONOMIC ANALYSIS 17
7.1 Overview and Perspective 17
7.2 Analysis population 18
7.3 Timing of analyses 18
7.4 Time Horizon 18
7.5 Analysis of costs & outcomes 18
7.6 Missing Data 19
7.7 Analysis of cost effectiveness 19
7.8 Sampling Uncertainty 20
7.9 Subgroup Analysis 20
7.10 Sensitivity analyses 20
7.11 Post-Trial Economic Modelling 21
7.12 Discounting 21
8. ANALYSIS OF SAFETY 22
8.1 Adverse events 22
9. REporting/publishing 23
9.1 Reporting Standards 23
9.2 Deviations from the SHEAP 23
10. PRIMARY REPORT TABLES AND FIGURES 24
11. REFERENCES 34
APPENDIX: Details of standard assessment tools 36

Abbreviations

CBT	Cognitive Behavioural Therapy
CFS	Chronic Fatigue Syndrome
CI	Confidence interval
CONSORT	Consolidated Standards of Reporting Trials
CPM	Counts per minute
GET	Graded Exercise Therapy
GP	General Practitioner
ITT	Intention to Treat
LPA	Light Physical Activity
MAGENTA	Managed Activity Graded Exercise in Teenagers and Pre-adolescents
MCID	Minimum Clinically Important Difference
ME	Myalgic Encephalomyelitis
MVPA	Moderate or Vigorous Physical Activity
NICE	National Institute for Health and Clinical Excellence
RCT	Randomised Controlled Trial
SCAS	Spence Children's Anxiety Scale
SD	Standard Deviation
SF-36-PFS	Short Form 36 Physical Function Subscale

1. INTRODUCTION & PURPOSE

This document details the rules proposed and the presentation that will be followed, as closely as possible, when analysing and reporting the results from MAGENTA.

The purpose of the plan is to:

1. Ensure that the analysis is appropriate for the aims of the trial, reflects good statistical practice, and that interpretation of a priori and post hoc analyses respectively is appropriate.
2. Explain in detail how the data will be handled and analysed to enable others to perform the actual analysis in the event of sickness or other absence.

Additional exploratory or auxiliary analyses of data not specified in the protocol are permitted but fall outside the scope of this analysis plan (although such analyses would be expected to follow Good Statistical Practice).

This analysis plan will be published on the University of Bristol online repository and therefore publicly available. Additional analyses suggested by reviewers or editors of journals will, if considered appropriate, be performed in accordance with the Analysis Plan, but if reported the source of such a post-hoc analysis will be declared.

Amendments to the statistical and health economics analysis plan will be described and justified in the final report of the trial.

2. SYNOPSIS OF STUDY DESIGN AND PROCEDURES

2.1 Trial objectives and aims

The overall aim of the study is to investigate the effectiveness and cost-effectiveness of Graded Exercise Therapy (GET) compared to Activity Management for the treatment of chronic fatigue syndrome/Myalgic Encephalomyelitis (CFS/ME) in children and adolescents.

2.1.1 Primary objective

Estimate the effectiveness of GET compared to Activity Management for paediatric CFS/ME.

2.1.2 Secondary (economic) objective

Estimate the cost-effectiveness of GET compared to Activity Management.

2.2 Trial design and configuration

A two parallel group randomised controlled trial, with internal pilot and feasibility work.

2.3 Trial centres

Specialist paediatric CFS/ME services in Bath, Newcastle and Cambridge.

2.4 Eligibility criteria

2.4.1 Inclusion criteria

- Age between 8 and 17 years inclusive.
- Diagnosis of CFS/ME (made using NICE guidance) at clinical assessment (NICE, 2007).

2.4.2 Exclusion criteria

- Severely affected by CFS/ME. NICE defines severe CFS/ME as individuals who are unable to do activity for themselves, or carry out minimal daily tasks only, or they have severe cognitive difficulties or depend on wheelchair for mobility (NICE, 2007).
- Have been referred for cognitive behavioural therapy (CBT) at their first clinical assessment.
- Unable to attend clinical / Skype sessions.

2.5 Description of interventions

Activity Management will be delivered by CFS/ME specialists (occupational therapists, physiotherapists, nurses, psychologists). All three services involved in MAGENTA provide Activity Management as routine, so no trial-specific training will be required. Activity Management aims to convert a "boom-bust" pattern of activity (lots one day and little the next) to a baseline with the same daily amount. For children with CFS/ME these are almost entirely cognitive activities: school, school-work, reading, socialising, and screen time (phone, laptop, TV, games). Those allocated to this treatment arm will receive advice about the total amount of daily activity, including physical activity, but will not receive specific advice about their use of exercise, increasing exercise or timed physical exercise.

Graded Exercise Therapy will be delivered by referral to CFS/ME specialists trained in the intervention who will receive guidance on the mandatory, prohibited and flexible components. Children and adolescents will be offered advice that is focused on exercise with detailed assessment of current physical activity, advice about exercise and a programme including timed daily exercise. Young people will be asked to record the amount of exercise. All participants receiving GET will be taught to take their heart rate to avoid overexertion. Children and adolescents in this group will not receive advice on cognitive activity, will not discuss the different types of cognitive activities, and will not be instructed to record cognitive activities.

Treatment delivery: In both treatment arms, children, their parents/carers and the clinician providing treatment will choose the number of follow up sessions (estimated to be between 8 & 12) and the frequency of appointments (every 2-6 weeks), in line with standard clinical practice. We will collect the number, frequency and length of follow up sessions for each participant as well as data on heart rate monitor use. Therapist may offer appointment via skype; these sessions will still be audio recorded. Children will be asked to provide consent to the audio recording of treatment session on paper forms, to be returned by post, or on-line via the REDCAP system.

2.6 Randomisation procedures

Once the participant has signed the consent/assent form and completed the clinical assessment (baseline research assessment), the study researcher will use the automated telephone/web randomisation service operated by the Bristol Randomised Trials Collaboration. Allocation to the two treatment arms of either GET or Activity Management (allocation ratio 1:1) will use minimisation to facilitate balance between trial arms by age (categories are 8-12, 13-17 years) and gender. We will retain a random component to prevent accurate prediction of allocation.

2.7 Blinding and breaking of blind

Because of the nature of the intervention it is not practical to blind either the family or the clinical service to treatment allocation. If allocation is done during the recruitment appointment, families are told the allocation immediately by the recruiting research nurse, who is not part of the treatment team. If the allocation is done later the recruiter will phone the family with their allocation. After allocation the recruiter will inform the clinical service who will then write to the young person/family with their appointment details. GPs will be told what intervention the young person will receive as part of routine clinical practice.

2.8 Trial committees

MAGENTA has an independent Trial Steering Committee and independent Data and Safety Monitoring Committee. Safety outcomes will be reviewed by the Data and Safety Monitoring Committee and reported to the Trial Steering Committee.

2.9 Outcome measures

2.9.1 Primary outcome

The primary outcome is the Short-Form 36 physical function sub-scale (SF-36-PFS) completed by the child at six months after randomisation (Ware 1993, Ware & Sherbourne 1992).

2.9.2 Secondary outcomes

Child self-completed questionnaires, at recruitment, and six and twelve months post-randomisation, measuring: school attendance (percentage of expected sessions); Chalder Fatigue score (Chalder 1993); pain visual analogue scale, Spence Children's Anxiety Scale (SCAS, Spence, Barrett & Turner 2003) and the Hospital Anxiety and Depression Scale (HADS, if they are 12-17 years old, Zigmond & Snaith 1983, White 1999). In addition, the SF-36_PS assessed at 12 months is also considered a secondary outcome. The Clinical Global Impression Scale is assessed at six and twelve months post-randomisation only. Questionnaires will be scored according to the authors' instructions, including the accommodation of items without a response.

Accelerometer measured physical activity for seven days within one month of randomisation and at three- and six-months follow-up. Accelerometer data will be processed using Kinesoft (v3.3.75; Kinesoft, Saskatchewan, Canada) in 60-s epochs, and will be included if participants provided at least three valid weekdays. A valid day was defined as at least 500 minutes (from 6:00am to 11:00pm), after excluding intervals of ≥60 minutes of zero counts allowing up to two minutes of interruptions. Mean minutes of

sedentary, light, moderate-to-vigorous (MVPA), and vigorous-intensity physical activity, were established using the Evenson accelerometer cut-off point (Evenson 2008). The mean accelerometer counts per minute, which provides an indication of the volume of physical activity in which the participant engages, will also be derived using the methods used in the International Children's Accelerometry Database (Cooper 2015).

Child/Adolescent self-completed questionnaires will be measuring health related quality of life (EQ-5D-Y) at baseline, 6 and 12 months. The EQ-5D-Y is a validated, generalised health profile questionnaire used to determine health related quality of life and deemed appropriate for use with children and adolescents up to 18 years old (Ravens-Sieberer 2010, Wille 2010). The questionnaire is self-completed by the young person. The EQ-5D-Y consists of a descriptive system with 5 dimensions (mobility, looking after myself, doing usual activities, having pain or discomfort, and feeling worried, sad or unhappy) each with 3 response levels, and a visual analogue scale (VAS) which asks participant to rate their current health status on a scale from 0 (worst health can imagine) to 100 (best health can imagine). At the study design stage, it was envisaged that utility scores would be derived from responses to the EQ-5D-Y using tariffs validated for use in UK adolescents to estimate QALYs over the 12-month period, adjusting for any imbalances in baseline EQ-5D-Y scores. However, the EuroQoL group has not published UK adolescent valuations for the EQ-5D-Y and subsequent research has indicated that valuations from the adult version of the questionnaire (EQ-5D-3L) are not valid for adolescents (Kind 2015). The implications of this for our analysis are discussed in section 7.

2.10 Economic data measurement and valuation

The following items of resource use will be measured: delivery of intervention (i.e. number of therapy sessions, number of minutes per therapy session), medications and other health services used by the child/adolescent (for example, GP visits, CBT sessions, medications), parent/carer productivity losses (for example, time off work, productivity loss, loss of earnings) and personal expenditure (for example, over the counter medications, travel expenses). For further detail see table 8.

Data regarding the delivery of the activity management and GET interventions, CBT or mental health service care use will be extracted from Millennium (the patient administrative database used by Royal United Hospitals Bath NHS Foundation Trust). Parent/Carers will self-complete questionnaires on child/adolescent use of medications and other health services parent/carers productivity and personal expenses at 6- and 12-

months follow-up. All resource use will be valued in monetary terms using appropriate UK unit costs or participant valuations estimated at the time of analysis.

Activity management and GET sessions will be valued using representative NHS band staff salaries, on costs, overheads and indirect costs detailed in the PSSRU Unit Costs of Health and Social Care (Curtis & Burns 2018), as will primary care consultations and other community-based health care services. Medication costs will be taken from the England Prescription Costs Analysis dataset (data.gov.uk) and British National Formulary (www.bnf.org). Costs directly reported in the Parent or Carer Questionnaires will be used to value out-of-pocket expenditures. Travel costs to attend appointments will be multiplied by the number of visits to estimate total travel costs for each participant. Changes in leisure time and loss of earnings due to work absences will be costed using the Office of National Statistics (ONS) Information on salaries.

2.11 Sample size and justification

The Minimum Clinically Important Difference (MCID) for the SF-36-PFS is 10 points (Brigden 2018) which is approximately 0.4 standard deviations (SD). For 80% power at 5% alpha, data on the primary outcome in 200 children is needed. Assuming a loss to follow-up of 10%, we need to recruit 222 children into the trial.

As there were no changes to trial procedures following the pilot trial conducted during the feasibility work, data from the 80 participants in the pilot will be included in the main analysis. Provision for this was made in the feasibility study protocol, and the informed consent procedures.

2.12 Interim analysis

No interim analyses of outcome measures by trial arm were conducted.

3. GENERAL ANALYSIS CONSIDERATIONS

3.1 Analysis populations

Full analysis set (for the primary and secondary analyses for the main results report): All randomised participants who complete the primary outcome measure, in the treatment group to which they were allocated. It is analysis of this population which is in accordance with the intention to treat (ITT) principle.

Accelerometer set: Participants will be included in the analysis of accelerometer data if they provide at least valid three weekdays (as defined in Section 2.9.2)

Safety set (for safety analyses): All randomised participants who received at least one session of their allocated intervention, in their allocated group.

3.2 Derived variables

Questionnaire measures: Item responses will be recorded using REDCap electronic data capture tools hosted at the University of Bristol (Harris 2009) and scale scores will be calculated within Stata.

Missing items in partially completed scales or subscales will be imputed using the methods described in the scale development literature where available (Bell 2016, Ware 1993).

Accelerometer measures: Each of the measures derived from accelerometer count data will be derived using dedicated software. Mean minutes of weekday light (LPA) and moderate to vigorous physical activity (MVPA) per day will be established using the threshold developed by Evenson and colleagues, which has been shown to be most accurate for this age group (Evenson 2008). The mean number of accelerometer counts per minute (CPM), which provides an indication of the volume of activity in which children engage, will be calculated.

3.3 Procedures for missing outcome data

The primary analysis will be based upon the observed data only. If primary outcome data are missing, one or more methods of adjusting for missing data will be employed in sensitivity analyses to explore how robust the observed primary analysis results are under different assumptions about the missing data mechanism.

3.4 Outliers

Outlying scores on the questionnaire measures are unlikely to be extreme enough to be overly influential on treatment effect estimates.

Outlying accelerometer counts can occur, e.g. a period of spuriously high counts which can occur during trampoline use, a cap of 11,714 counts per minute will be applied to the data (Rich 2014). Periods of 60 minutes or longer with zero counts will be defined as accelerometer non-wear time.

3.5 Visit windows

The baseline data collection takes place prior to random allocation.

The six-month follow-up questionnaires needed to be returned between six weeks before or up to three months after the exact date (183 days post-randomisation).

The twelve-month follow-up questionnaires needed to be returned between three months before or after the exact date (365 days post-randomisation).

The number of returned questionnaire packs returned but falling outside of these windows will be noted but treated as missing.

3.6 Software

Data analyses will primarily be carried out using Stata version 15.1 (StataCorp, College Station, Texas, USA, 2017). The construction of bootstrapped CE planes and CEACs will be conducted in Microsoft Excel with required coding in Visual Basic for Applications (VBA).

4. DESCRIPTION OF PARTICIPANT CHARACTERISTICS

4.1 Disposition

A flow of patients through the trial will be summarised in a CONSORT diagram that will include the eligibility, reasons for exclusion, numbers randomised to the two treatment groups, losses to follow up and the numbers analysed.

4.2 Baseline characteristics

Continuous data will be summarised in terms of the mean, standard deviation, and number of observations. Categorical data will be summarised in terms of frequency counts and percentages.

5. ASSESSMENT OF STUDY QUALITY

5.1 Eligibility checks

Eligibility assessments will be carried out by a specialist clinician during the initial clinical assessment and will be checked and confirmed by the recruiting research nurse.

5.2 Data validation

Data are collected into REDCap data capture system (Harris 2009) with range checks for variables. Baseline data will be collected on paper questionnaires in clinic and the follow-up questionnaires are sent via email using the automatic survey system within REDCap or by post if they do not have internet access.

5.3 Intervention fidelity and adherence

We will record the number of booked treatment sessions where the participant did not arrive or where there was a late cancellation (within 24 hours). We will assume that those who did not attend (or cancelled within 24 hours) three or more consecutive appointments or 50% of appointments did not find the intervention acceptable.

A tick-box checklist will be completed for each session by the therapist to assess fidelity to the mandatory and prohibited of the allocated intervention.

5.4 Protocol deviations

Protocol deviations which may affect the estimation of the treatment effect will be recorded and reported in the main study reports. Examples include a participant being found to be ineligible after random allocation, and a participant moving from their allocated treatment to the other study treatment within six months of allocation.

5.5 Changes made to the planned statistical analyses

The planned statistical analysis is described in this Statistical & Health Economics Analysis Plan, which has been written by co-investigators who have not had sight of the study data, and which will be signed and made public ahead of the analysis proceeding. Changes to the plan will be highlighted and justified in a revised version of the Statistical & Health Economics Analysis Plan.

Changes to the pre-specified analysis, the need for which is recognised during the analysis, will be highlighted in study reports and publications, and fully justified.

6. ANALYSIS OF EFFECTIVENESS

6.1 Primary analysis

The null hypothesis to be tested is that the population mean SF-36-PFS score at six months follow-up is equal between groups allocated to Graded Exercise Therapy or to Activity Management. This null hypothesis will be tested in an intention-to-treat analysis, which will compare study participants who completed the required measures, in the treatment groups to which they were allocated (the full analysis population). The treatment effect will be estimated as a difference between sample means, which will be presented with 95% confidence interval and p-value.

The difference in means will be estimated in a linear regression model with patient response at six months post-randomisation (y_i) as the outcome variable and covariates: treatment allocation (x_{1i}=1: GET; x_{1i}=0: activity management), baseline SF-36-PFS (x_{2i}), age at recruitment (x_{3i} as a continuous measure), and gender (x_{4i}=1: male; x_{4i}=0: female). Finally a dummy variable distinguishing those participant without a baseline assessment of outcome (x_{5i}=1: no baseline assessment; x_{5i}=0: baseline assessment available). A normal distribution is assumed for the residual errors: e_i~N(0,σ_e). The coefficient for the treatment allocation covariate (β_1) is the intention to treat estimate of treatment effectiveness, comparing activity management to GET. In statistical notation:

$$y_i = \beta_0 + \beta_1 x_{1i} + \beta_2 x_{2i} + \beta_3 x_{3i} + \beta_{4t} x_{4i} + \beta_5 x_{5i} + e_i$$

The residuals from the model will be checked for a normal distribution, and as having a similar standard deviation in the two treatment groups. If the model assumptions are violated, the confidence interval will be determined using a bias corrected and accelerated bootstrap method, and the p-value will be determined using a permutation test.

As nearly all participants were recruited at the Bath centre, there is no need to distinguish study centres in the analysis.

6.2 Secondary analyses

The primary analysis will be adapted to each of the other questionnaire measures (Chalder Fatigue Score, Pain VAS, Spence Children's Anxiety Scale, Hospital Anxiety and Depression Scales) and accelerometer measures (counts per minute, daily minutes of light physical exercise, daily minutes of moderate or vigorous physical exercise) at first and second follow-up assessments in turn (with the twelve-month assessment of SF-36-PF included as a secondary outcome). The corresponding baseline measure of the questionnaire being analysed will be included.

The primary analysis will also be adapted to the Clinical Global Impression Scale, with an ordered logistic regression model being employed. The seven response categories will be kept separate when included in this model. There is no baseline assessment of this measure (Section 9.1).

6.3 Sensitivity analysis

For the primary outcome we will conduct sensitivity analyses in which we further adjust our primary analysis model for prognostic variables for which there is a baseline imbalance between intervention arms (more than half a standard deviation between means, more than 0.1 between proportions). In addition, the number of days post randomisation on which the primary outcome measure was completed will be included as a covariate in this model.

For the primary outcome only we will conduct sensitivity analyses in which we will impute missing data to evaluate the robustness of the results and conclusions under different assumptions about how the missing data came about.

Summary statistics for the primary outcome will be presented by allocated intervention group, separately for those participants who were able to attend no more than two sessions, and more than two sessions.

6.4 Sub-group analyses

Due to weak evidence of a difference in the treatment effect between males and females in the SMILE trial (Crawley 2018), we will test the null hypothesis of no difference in the relative effects of activity management and GET on SF-36-PFS at six months post-randomisation between males and females. This null hypothesis will be tested by adding a treatment-gender interaction term to the regression model in Section 6.2.

7. HEALTH ECONOMIC ANALYSIS

7.1 Overview and Perspective

The health economic analysis will consist of a within-trial analysis of the cost-effectiveness of GET compared to activity management. The primary health economic analysis will be assessed from a UK NHS and personal social services perspective as recommended by NICE (NICE 2013). This will include the cost of contacts with primary care, secondary care, and medications. Wider societal costs outside of the healthcare sector, including loss of earnings, and opportunity costs incurred by family members will not be included within the primary analysis; these data will be collected and evaluated separately. Table A summarises the methods used in the economic evaluation:

Table A: Summary of Methods used in Health Economic Analysis

**Aspect of Methodology**	**Strategy for Base-Case**	**Alternative Strategy for Sensitivity Analysis**
Data set	All randomised participants, analysed on intention-to-treat (ITT) basis	Complier average causal effect
Primary Outcome	SF-36-PFS at 6-months	SF-36-PFS at 12-months
Secondary Outcome	EQ-5D-Y (administered at baseline, 6-months, and 12-months) - descriptive analysis of responses	EQ-5D-Y (administered at baseline, 6-months, and 12-months) – mapping descriptive system responses to most appropriate valuation set (e.g. UK EQ5D-3L value set or non-UK EQ5D-Y value set if and when available)
Costs included in analysis	Intervention cost<br>Health and personal social services costs including primary care, secondary care, and medication costs<br>All other healthcare costs are excluded	As for base-case with inclusion of wider societal costs including loss of earnings, and opportunity costs incurred by family members
Missing data	Multiple imputation	Available case analysis conducted on observed data
Adjustment for baseline covariates	Regression used to adjust outcomes for differences at baseline. Adjusting for baseline covariates and any stratification variables.	

7.2 Analysis population

All randomised participants who complete the primary outcome measure and have complete NHS cost data, will be analysed according to the treatment group to which they were allocated in accordance with the intention to treat (ITT) principle.

Missingness in costs and effects may be predictive of the values; failing to impute these values may result in analysis bias. To account for missingness, imputation will be employed in the primary economic analysis.

7.3 Timing of analyses

The primary analysis will be conducted once all patients have been followed for one year after randomisation.

7.4 Time Horizon

The primary economic analysis will be conducted at 6-months commensurate with the primary analysis. A secondary analysis will consider the longer-term treatment effect of GET compared to activity management assessed at 12-month follow-up.

7.5 Analysis of costs & outcomes

Resources utilised and associated costs will be summarised and tabulated in order to compare the total cost of GET compared to activity management. The delivery cost of GET and activity management will include the costs of staff time and any materials used estimated from the actual number of sessions participants attended.

Health care resource use will also be evaluated. Primary care contacts and impact on parent/carer work and resource use will be extracted from questionnaire responses. Hospital admissions will be obtained from Millennium. Resource usage costs will also be summarised and tabulated by contact type (e.g. A&E visits/admissions, GP visits, other primary or community care contacts.) Similarly, parent/carer costs (e.g. caregiver time off work, caregiver loss of earnings, over-the-counter medications) will be summarised and tabulated. The mean cost and variation (e.g. standard deviation) in each arm will be described by contact type (see Table 7). Differences in the use of services between treatment groups will be described but not compared statistically. Differences in overall mean costs between treatment groups will be analysed using appropriate regression techniques, with adjustment for relevant covariates.

The primary outcome for the health economic analysis will consist of a cost-effectiveness analysis conducted on the MCID on the SF-36-PFS. We will estimate the additional cost per patient achieving an MCID improvement of GET versus activity management, in line with NICE Guide to the Methods for Technology Appraisal (NICE 2013).

Descriptive statistics for the EQ-5D-Y™ will be tabulated. As described above, value sets for a UK/England based youth population have not yet been published; in secondary exploratory analysis mapping will be conducted to either English adult population values or available youth population values from an alternative country and utility scores at 6 and 12 months will be compared between treatment arms. Descriptive analysis of responses from the EQ-5D-Y™ VAS will be presented.

7.6 Missing Data

Missing baseline data will be handled commensurate with the statistical analysis.

Any missing items in the EQ-5D-Y™ questionnaire will not be individually imputed. Instead, utility values will be imputed in accordance with unit non-response procedures (i.e. available case analysis or multiple imputation.)

The structure of the resource use questionnaire implies that data will either be fully complete or fully incomplete. Missing data will be imputed in accordance with unit non-response procedures (available case analysis or multiple imputation).

The pattern of missing outcome data, after implementation of item-non-response procedures outlined above, will be examined. For the base case health economic analysis, data will be assumed missing at random (MAR) (missingness dependent upon observed data but not the unobserved outcomes), and multiple imputation using chained equations will be used to impute missing items to provide an unbiased analysis on all randomised individuals. Imputations will be combined following Rubin's rules (Rubin, 1987). Due to the predefined range of outcome scores, including utility derived from the EQ-5D-Y™, predictive mean matching (PMM) will be used to ensure imputed values are consistent with observed data. The plausibility of the MAR assumption will be explored by comparing observed data in participants with and without the item of interest and further sensitivity analyses will explore the robustness of conclusions should outcomes be assumed to be missing not at random (MNAR).

7.7 Analysis of cost effectiveness

Cost-effectiveness analysis will be used to estimate the cost per MCID in the SF-36-PFS based on the primary endpoint at 6-months. Incremental costs and effects will be presented in a disaggregated format with associated 95% confidence intervals. Seemingly Unrelated Regression (SUR) will be used if appropriate to account for the correlation between the costs and effects.

The comparative analysis of incremental costs and effects will be summarised in terms of an incremental cost-effectiveness ratio (ICER). The ICER can be represented as:

$$ICER = \frac{\Delta C}{\Delta E} = \frac{C_1 - C_0}{E_1 - E_0}$$

Where C_1 and E_1 are the costs and effects of GET (the intervention group), and C_0 and E_0 are the cost and effects of activity management (the usual care group); with ΔC and ΔE the incremental costs and effects associated with the intervention compared to usual care. Where simple dominance of the intervention is observed e.g. the intervention is less costly and more effective than usual care, ICERs will not be presented. The final report will show sufficient information to reproduce the calculations of the ICER.

7.8 Sampling Uncertainty

The ICER is reported to determine the cost-effectiveness of the intervention compared to competing alternatives and aid decision making.

To explore uncertainty regarding the costs and effects, non-parametric bootstrapping will be undertaken to analyse the joint uncertainty of parameter estimates. A minimum of 5,000 resamples will be performed. Results will be illustrated using a cost-effectiveness plane (Figure 2).

The probability of cost-effectiveness will be depicted using a cost-effectiveness acceptability curve (CEAC) across a range of willingness-to-pay (WTP) thresholds. At a range of feasible WTP thresholds, the probability of cost-effectiveness will be explicitly highlighted. The reported ICERs from our analysis are presented to assist the decision-making process and are not an absolute statement on whether the intervention can be deemed cost-effective.

7.9 Subgroup Analysis

Subgroup analysis will be performed commensurate to those specified in section 6.4.

7.10 Sensitivity analyses

Several sensitivity analyses will be undertaken to explore key uncertainties surrounding key parameters in the economic evaluation. Deterministic one-way sensitivity analyses will be undertaken to examine the impact of changes in key parameters on the ICER e.g. upper/lower 95% CIs, different assumptions regarding intervention costs. A series of sensitivity analyses will investigate different approaches to the issue of missing data. Additional sensitivity analyses may be added. Any that are added after the initial unblinded analysis will be clearly labelled as post-hoc analyses.

7.11 Post-Trial Economic Modelling

No health economic modelling is planned in this study. Future work may wish to investigate the longer-term costs and effects of GET compared to AM.

7.12 Discounting

As the study period of the trial does not exceed 12-months, no discounting will be applied to the within-trial analysis

8. ANALYSIS OF SAFETY

8.1 Adverse events

We will define serious adverse events as "any adverse event that results in death, is life threatening, requires hospitalisation or prolongation of existing hospitalisation, results in persistent or significant disability or incapacity.

We will define a serious deterioration in health as either a decrease of 20 or more in SF-36-PFS score between baseline and six- or twelve-month follow-up, scores of "much" or "very much worse" on the Clinical Global Impression scale at 6 or 12 months or withdrawal from treatment because of feeling worse.

All adverse events will be recorded in detail on a case record form. At the conclusion of the study and during the safety analyses, all adverse events recorded during the study will be presented in the final study report.

Data on adverse events will be collected for each participant from the point at which they consent to take part in the study until the end of the follow-up period (12 months).

9. REPORTING/PUBLISHING

9.1 Reporting Standards

Reporting of the MAGENTA methodology and results will follow the CONSORT guidelines, including the extension for non-pharmacological treatments (www.consort-statement.org/).

CHEERS guidelines will be followed when reporting the health economic evaluation, in a format appropriate to stakeholders and policy makers (Husereau 2013).

9.2 Deviations from the SHEAP

Any deviation from the SHEAP will be described and justified in the final published report

10. PRIMARY REPORT TABLES AND FIGURES

Table 1. Characteristics of the randomised participants at baseline

	**Graded Exercise Therapy (n=)**		**Activity Management (n=)**	
Mean age (SD)				
Number female (%)				
Median months since illness onset (25th, 75th percentiles)				
Number comorbid anxiety[1] (%)				
Number comorbid depression[1] (%)				
Mean SF-36 Physical Function score (SD)				
Mean Chalder Fatigue score (SD)				
Mean pain VAS (SD)				
Mean SCAS (SD)				
Mean HADS anxiety score (SD)				
Mean HADS depression score (SD)				
School attendance in the previous week[2]:				
Number 0 days (%)				
Number 0.5 days (%)				
Number 1 day (%)				
Number 2 days (%)				
Number 3 days (%)				
Number 4 days (%)				
Number 5 days (%)				

1. Determined using the K-SADS.
2. Data has been collected on the number of hours of home tuition; this will be reported in the text accompanying this table.

Table 2. Treatment fidelity and adherence

	**Graded Exercise Therapy (n=###)**	**Activity Management (n=###)**
Number of patients with one or more sessions recorded as not including a compulsory element or including a prohibited element (%)		
Mean (SD) sessions of allocated therapy		
Number not starting (one or no sessions attended) allocated treatment (%)		
Number not completing six months of allocated treatment (%)		
Number starting non-allocated treatment within six months of randomisation (%)		
Number starting non-allocated treatment within twelve months of randomisation (%)		

Table 3. Summary statistics and treatment effect estimates for the Short Form 36 physical function at 6- (primary outcome measure) and 12-months

	Graded Exercise	Activity Management	Difference in means (95% CI)	p-value
	**Mean (SD), N**	**Mean (SD), N**		
Primary analysis (6 months)				
Sensitivity analysis (6 months, further covariates[1])				
Sensitivity analysis (6 months, missing data imputed)				
Sensitivity analysis (6 months, participants attending ≤2 sessions)				
Sensitivity analysis (6 months, participants attending 3+ sessions)				
Observed data (12 months)				
	**n (%)**	**n (%)**		
Decrease of 20+ points on the SF-36-PFS at any post-baseline assessment			N/A	N/A

1. Covariates added for measures not balanced at baseline, and for exact time of primary outcome completion

Table 4. Summary statistics and treatment effect estimates for the secondary outcome questionnaire measures at 6 months and 12 months

	Graded Exercise	Activity Management	Difference in means (95% CI)	p-value
	Mean (SD), N	Mean (SD), N		
Chalder Fatigue 6 months				
Chalder Fatigue 12 months				
Pain VAS 6 months				
Pain VAS 12 months				
SCAS 6 months				
SCAS 12 months				
HADS anxiety 6 months				
HADS anxiety 12 months				
HADS depression 6 months				
HADS depression 12 months				
School attendance 6 months*				
School attendance 12 months*				

*As a proportion of full time

Table 5. Summary statistics and treatment effect estimates for the secondary outcome accelerometer measures at 6 months and 12 months.

	Graded Exercise	Activity Management	Difference in means (95% CI)	p-value
	Mean (SD), N	Mean (SD), N		
Counts per minute baseline				
Counts per minute 3 months				
Counts per minute 6 months				
Daily minutes:				
Sedentary baseline			--	
Sedentary 3 months				
Sedentary 6 months				
Light physical activity baseline			--	
Light physical activity 3 months				
Light physical activity 6 months				
Moderate-to-vigorous physical activity baseline			--	
Moderate-to-vigorous physical activity 3 months				
Moderate-to-vigorous physical activity 6 months				
Vigorous physical activity baseline			--	
Vigorous physical activity 3 months				
Vigorous physical activity 6 months				

Table 6. Participant-rated Clinical Global Impression Scale of change in overall health from baseline

	**Graded Exercise**	**Activity Management**	**Odds ratio (95% CI)**	**p-value**
Change from baseline (6 months)				
Much better or very much better (%)				
Minimal change (%)[1]				
Much worse or very much worse (%)				
Change from baseline (12 months)				
Much better or very much better (%)				
Minimal change (%)				
Much worse or very much worse (%)				

1. Includes the responses "no change", "a little better", and "a little worse".

Table 7 Example of how resource use may be presented

**Resource category**	**GET N= XX**			**AM N=XX**		
	**N**	**Mean resource use (SD)**	**Mean cost (£) (SD)**	**N**	**Mean resource use (SD)**	**Mean cost (£) (SD)**
**NHS Costs**						
GET Sessions						
AM Sessions						
Other outpatient appointments						
A&E visits/admissions						
GP visits						
GP nurse visits						
GP phone calls						
GP home visits						
Contacts with school counsellor						
Nurse at walk-in centre						
NHS Direct						
Other primary or community care contacts						
Prescriptions						
Total NHS costs						
**Parent/Carer Costs**						
Time off work - Caregiver 1						
Time off work - Caregiver 2						
Productivity – Caregiver 1						
Productivity – Caregiver 2						
Daily activities– Caregiver 1						
Daily activities– Caregiver 2						
Hospital travel						
Primary or community care travel						
Over-the-counter medication						
Loss of earnings – Caregiver 1						
Any other costs						
Total Societal costs						

Table 8. Example of how the main economic analyses may be presented

**Allocation arm**	**N**	**Adjusted cost (£) mean (95% CI)**	**Adjusted QALY mean (95% CI)**	**Incremental cost (£) (95% CI)**	**Incremental QALY (95% CI)**	**ICER (£/QALY)**	**NMB (£) at £20000/QALY (95% CI)**
NHS Costs							
GET							
AM							
Parent/Care Giver Costs							
GET							
AM							
Total Costs							
GET							
AM							

Table 9. Example of how the economic sensitivity analyses maybe presented

**Allocation arm**	**N**	**Adjusted cost (£) mean (95% CI)**	**Adjusted QALY mean (95% CI)**	**Incremental cost (£) (95% CI)**	**Incremental QALY (95% CI)**	**ICER (£/QALY)**	**NMB (£) at £20000/QALY (95% CI)**
Sensitivity Analyses 1: Different ways of dealing with missing data							
GET							
AM							
Sensitivity Analyses 2: Complete Case/Imputed							
GET							
AM							
Sensitivity Analyses 3: XXX							
GET							
AM							
Sensitivity Analyses 4: XXX							
GET							
AM							

(Above are examples, repeat as many times number of sensitivity analyses performed)

Figure 1. CONSORT recruitment and retention flow chart

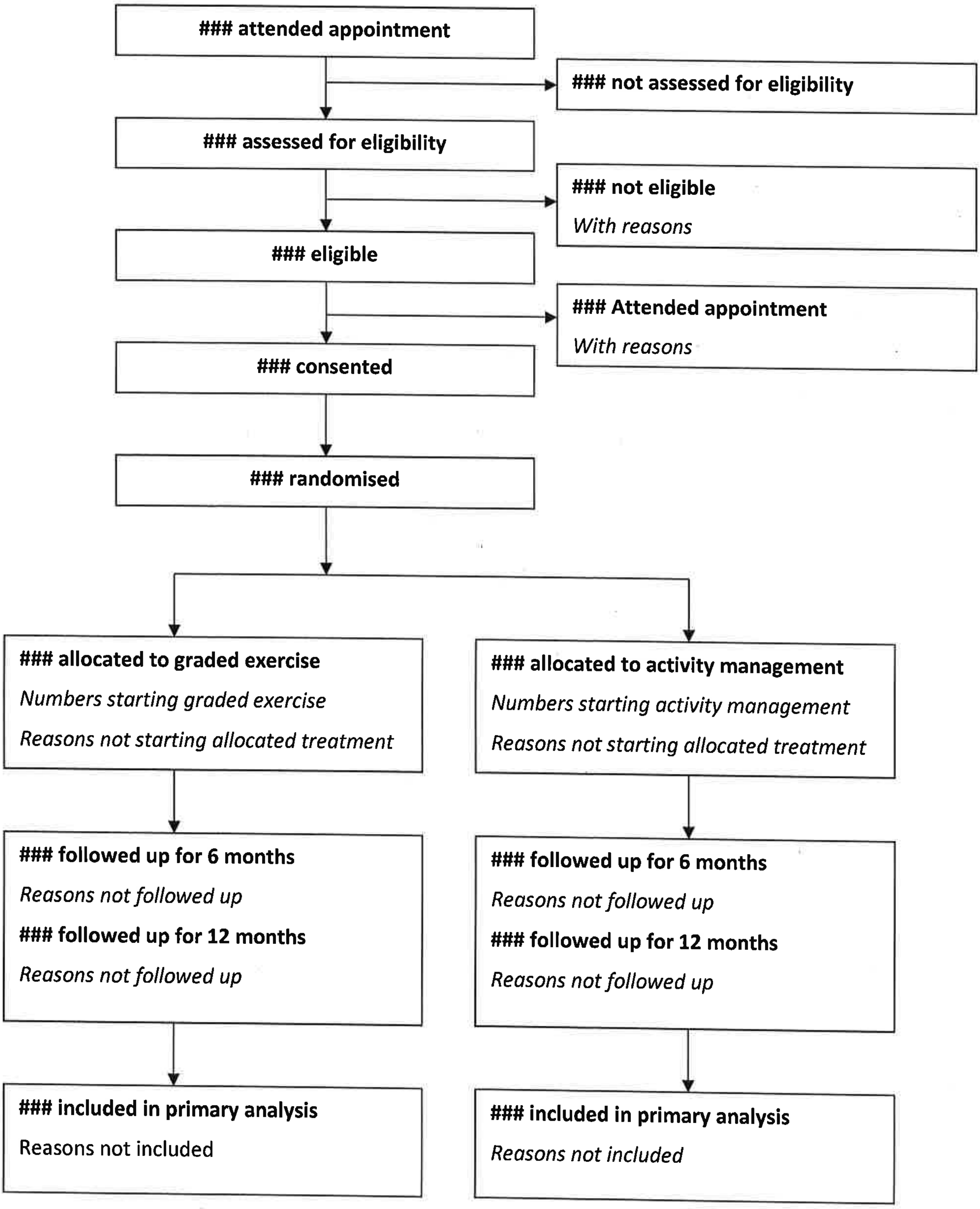

Figure 2: Example Cost-effectiveness plane

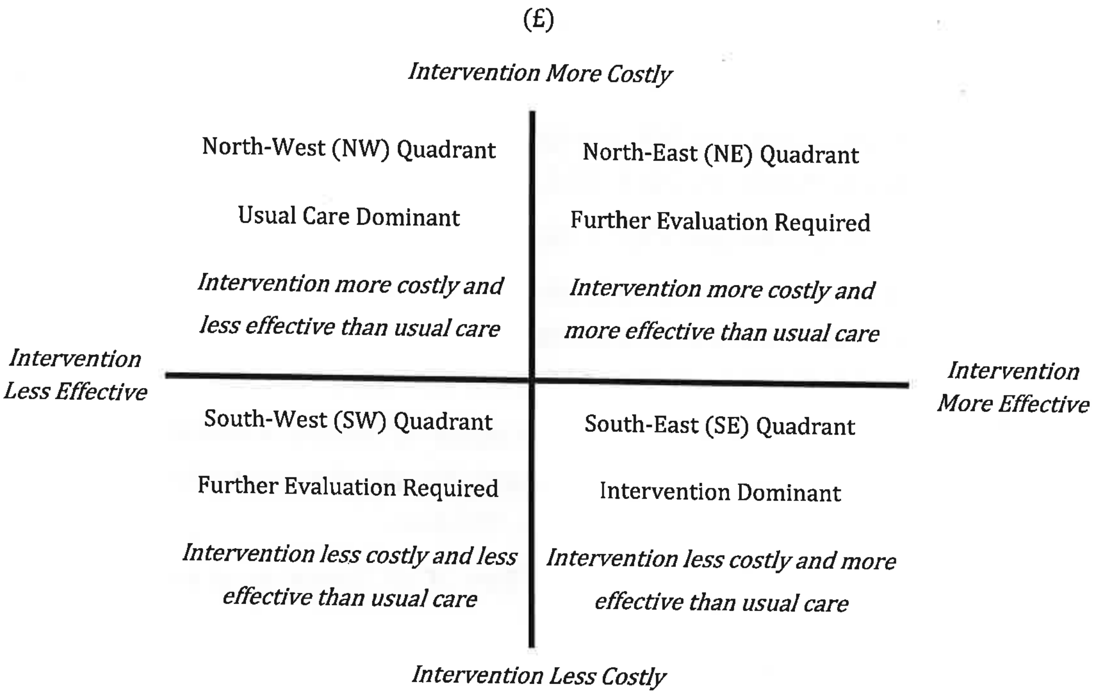

Figure 3: Example Cost-effectiveness acceptability curve

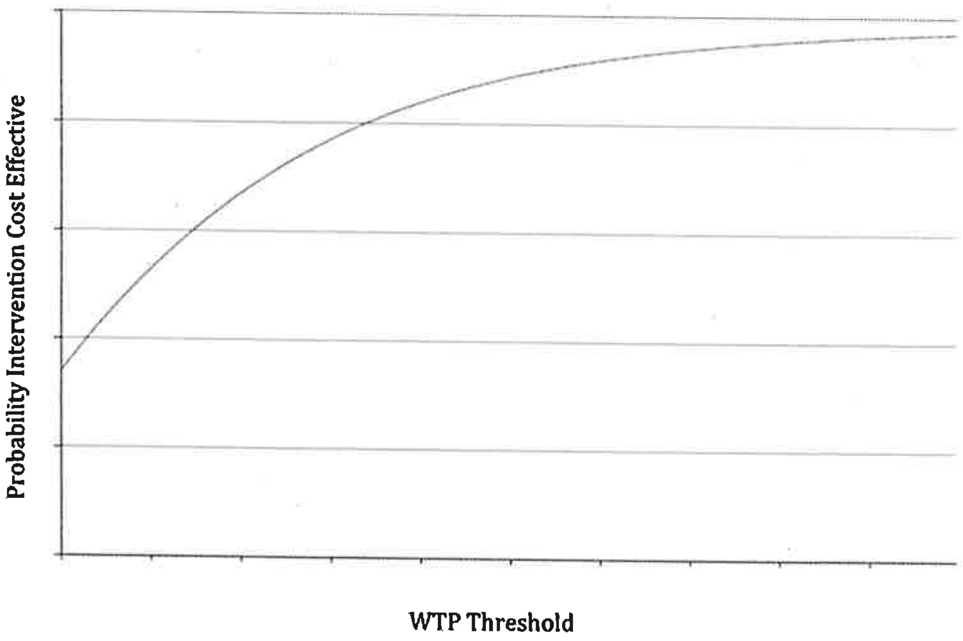

11. REFERENCES

Bell, M.L., et al., *Handling missing items in the Hospital Anxiety and Depression Scale (HADS): a simulation study.* BMC research notes, 2016. **9**(1): p. 479-479.

Brigden, A., et al., *Defining the minimally clinically important difference of the SF-36 physical function subscale for paediatric CFS/ME: triangulation using three different methods.* Health and quality of life outcomes, 2018. **16**(1): p. 202-202.

Chalder, T., et al., *Development of a fatigue scale.* J Psychosom Res, 1993. **37**(2): p. 147-53.

Cooper, A.R., et al., *Objectively measured physical activity and sedentary time in youth: the International children's accelerometry database (ICAD).* The international journal of behavioral nutrition and physical activity, 2015. **12**: p. 113-113.

Crawley, E.M., et al. *Clinical and cost-effectiveness of the lightning process in addition to specialist medical care for paediatric chronic fatigue syndrome: Randomised controlled trial.* Archives of Disease in Childhood, 2018. 103(2): p. 155-164.

Curtis, L.A. and Burns, A. *Unit Costs of Health and Social Care 2018.* University of Kent, Canterbury, UK. 2018.

Evenson, K.R., et al., *Calibration of two objective measures of physical activity for children.* J Sports Sci, 2008. **26**(14): p. 1557-1565.

Harris, P.A., et al., *Research electronic data capture (REDCap)--a metadata-driven methodology and workflow process for providing translational research informatics support.* Journal of biomedical informatics, 2009. **42**(2): p. 377-381.

Hawker, G.A., et al., *Measures of adult pain: Visual Analog Scale for Pain (VAS Pain), Numeric Rating Scale for Pain (NRS Pain), McGill Pain Questionnaire (MPQ), Short-Form McGill Pain Questionnaire (SF-MPQ), Chronic Pain Grade Scale (CPGS), Short Form-36 Bodily Pain Scale (SF-36 BPS), and Measure of Intermittent and Constant Osteoarthritis Pain (ICOAP).* Arthritis Care Res (Hoboken), 2011. **63 Suppl 11**: p. S240-52.

Husereau D., et al. *Consolidated health economic evaluation reporting standards (CHEERS) statement.* BMC Medicine, 2013, **11**:80.

Kind P, Klose K, Gusi N, Olivares PR, Greiner W. *Can adult weights be used to value child health states? Testing the influence of perspective in valuing EQ-5D-Y.* Quality of Life Research, 2015, **24:** 2519-39.

NICE, *Chronic fatigue syndrome/myalgic encephalomyelitis (or encephalopathy): Diagnosis and management of CFS/ME in adults and children (NICE guidelines CG53).* 2007: London.

NICE. *Guide to the Methods of Technology Appraisal.* 2013. Available: https://www.nice.org.uk/process/pmg9/chapter/the-reference-case#evidence-on-resource-use-and-costs Accessed 23/07/2019.

Ravens-Sieberer, U., et al. Feasibility, reliability, and validity of the EQ-5D-Y: results from a multinational study. Quality of Life Research, 2010. 19(6): p. 887-897.

Rich, C., et al., *Quality control methods in accelerometer data processing: identifying extreme counts.* PloS one, 2014. **9**(1): p. e85134-e85134.

D. B. Rubin, Multiple Imputation for Nonresponse in Surveys, New Jersey: John Wiley & Sons, 1987.

Spence, S.H., P.M. Barrett, and C.M. Turner, *Psychometric properties of the Spence Children's Anxiety Scale with young adolescents.* J Anxiety Disord, 2003. **17**(6): p. 605-25.

Ware, J.E., *SF-36 Health Survey Manual and Interpretation Guide* 1993, The Health Institute, New England Medical Center Boston, Massachusetts

Ware, J.E. and C.D. Sherbourne, *The MOS 36-item short-form health survey (SF-36). I. Conceptual framework and item selection.* Med Care, 1992. **30**(6): p. 473-83.

White, D., et al., *Validation of the Hospital Anxiety and Depression Scale for use with adolescents.* Br J Psychiatry, 1999. **175**: p. 452-4.

White, P.D., et al., *Comparison of adaptive pacing therapy, cognitive behaviour therapy, graded exercise therapy, and specialist medical care for chronic fatigue syndrome (PACE): a randomised trial.* Lancet, 2011. **377**(9768): p. 823-36.

Wille, N., et al. Development of the EQ-5D-Y: a child-friendly version of the EQ-5D. Quality of Life Research, 2010. 19(6): p. 875-86.

Zigmond, A.S. and Snaith R.P. *The hospital anxiety and depression scale.* Acta Psychiatrica Scandinavica, 1983. **67**(6): p. 361-70.

APPENDIX: DETAILS OF STANDARD ASSESSMENT TOOLS

Chalder Fatigue Scale (Chalder 1993): A self-completed 14-item measure of fatigue, with four response options per question: "better than usual" (score 0), "no more than usual" (score 1), "worse than usual" (score 2) and "much worse than usual" (score 3). Items include "Do you have problems with tiredness?" and "Do you have difficulty concentrating?". The range of scores is 0 to 42, with higher scores being most fatigue.

Short Form 36 Physical Function (Ware 1993, Ware & Sherbourne, 1992): A self-completed 10-item sub-scale of the Short Form 36. Response options are "Yes, limited a lot", "Yes, limited a little" and "No, not limited at all". The range of scores is 0 to 100, with higher scores being the best function.

Hospital Anxiety and Depression Scale (Zigmond & Snaith 1983, White 1999): A self-completed 14-item questionnaire, with anxiety (7 items) and depression (7 items) subscales. Example items include "I feel tense or wound up" and "I still enjoy the things I used to enjoy", with four response options scored 0 to 3. The range of scores for each sub-scale is 0 to 21, with higher scores indicating a more depressed mood or anxious state. Only used for children aged 12 and above.

Spence Children's Anxiety Scale (Spence, Barrett & Turner, 2003): A self-completed measure of anxiety, consisting of 44 items, 38 of which assess specific anxiety symptoms relating to six sub-scales, and six of which are positive filler items aimed at reducing negative response bias. Respondents are asked to indicate the frequency with which each occurs on a four-point scale ranging from "Never" (score 0) to "Always" (score 3). A total SCAS score is obtained by summing the scores of the 38 anxiety items, hence a range from 0 to 114, with higher scores indicating greater anxiety.

Pain Visual Analogue Scale (Hawker 2011): The respondent places a line perpendicular to the 100mm long VAS line at the point that represents their pain intensity. The score is determined by measuring the distance from the "no pain" anchor to the respondent's mark. The range of scores is 0 to 100, with higher scores indicating greater pain intensity.

Clinical Global Impression Scale (White 2011): Participant completed, assessing change from baseline with seven response categories. Here we follow White and colleagues in grouping the response categories into negative change ("Very much worse" or "Much worse"), minimal change ("A little worse", "No change" or "A little better"), and positive change ("Much better" or "Very much better").
